# Supplementary material for: Interfacial assembly of binary atomic metal-Nx sites for high-performance energy devices
Source: Nat Commun. 2023 Apr 1;14:1822. doi: 10.1038/s41467-023-37529-2 (PMC10067952; doi:10.1038/s41467-023-37529-2)
Supplement: Supplementary file 1 — Supplementary Information [file 41467_2023_37529_MOESM1_ESM.pdf]

Supplementary Information

Interfacial Assembly of Binary Atomic Metal-N_x Sites for High-Performance Energy Devices

Zhe Jiang[1,2,3,#], Xuerui Liu[4,#], Xiao-Zhi Liu[5,#], Shuang Huang[6], Ying Liu[1], Ze-Cheng Yao[1,3], Yun Zhang[1], Qing-Hua Zhang[5], Lin Gu[5], Li-Rong Zheng[7], Li Li[6], Jianan Zhang[8], Youjun Fan[2,*], Tang Tang[1,3,*], Zhongbin Zhuang[4], Jin-Song Hu[1,3,*]

[1] Beijing National Laboratory for Molecular Sciences (BNLMS), Institute of Chemistry, Chinese Academy of Sciences, Beijing 100190, China.
[2] Guangxi Key Laboratory of Low Carbon Energy Materials, School of Chemistry and Pharmaceutical Sciences, Guangxi Normal University, Guilin 541004, China.
[3] University of Chinese Academy of Sciences, Beijing 100049, China.
[4] Beijing Advanced Innovation Center for Soft Matter Science and Engineering, Beijing University of Chemical Technology, Beijing 100029, China.
[5] Beijing National Laboratory for Condensed Matter Physics, Institute of Physics, Chinese Academy of Sciences, Beijing 100190, China.
[6] Chongqing Key Laboratory of Chemical Process for Clean Energy and Resource Utilization, School of Chemistry and Chemical Engineering, Chongqing University, Chongqing 400044, China.
[7] Beijing Synchrotron Radiation Facility, Institute of High Energy Physics, Chinese Academy of Sciences, Beijing 100049, China.
[8] College of Materials Science and Engineering, Zhengzhou University, Zhengzhou, 450001 China.

[#] These authors contributed equally: Zhe Jiang, Xuerui Liu, and Xiao-Zhi Liu

Supplementary Figures

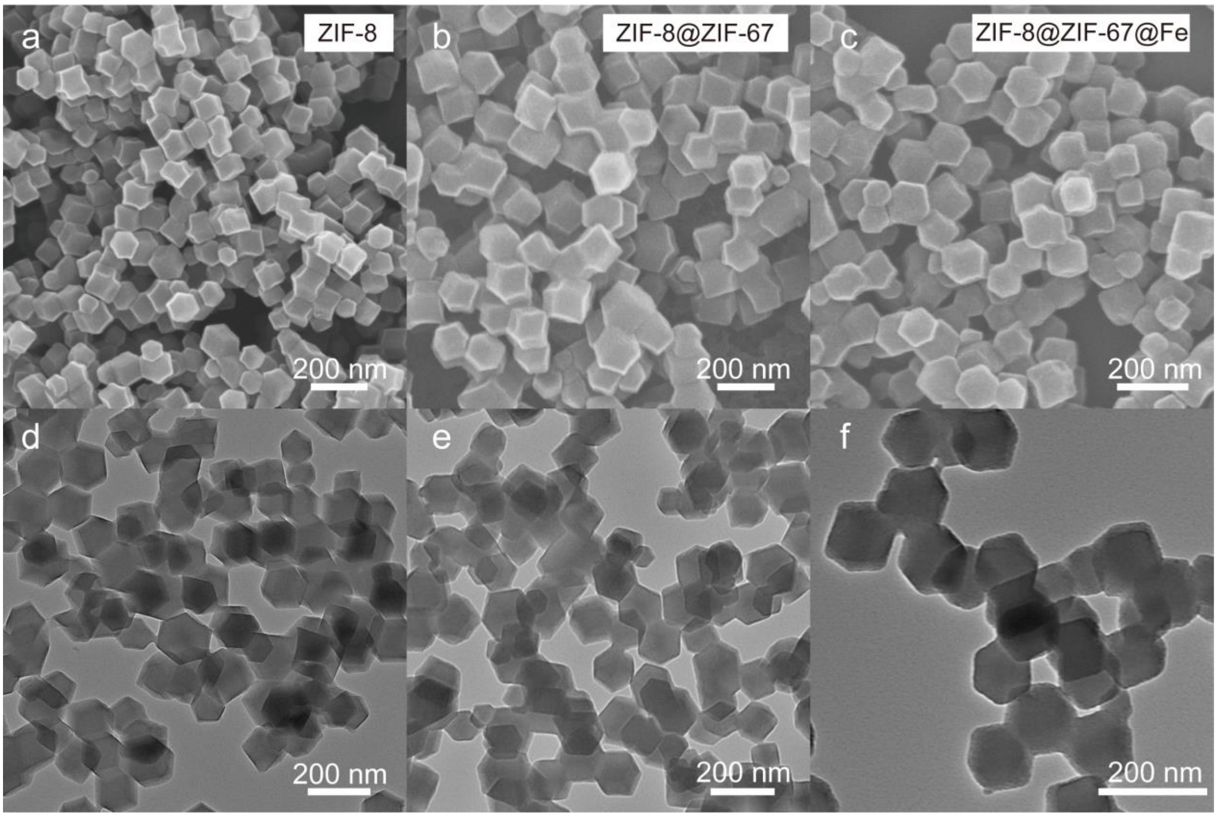

Supplementary Figure 1. SEM and TEM images of (a,d) ZIF-8, (b,e) ZIF-8@ZIF-67, and (c,f) ZIF-8@ZIF-67@Fe.

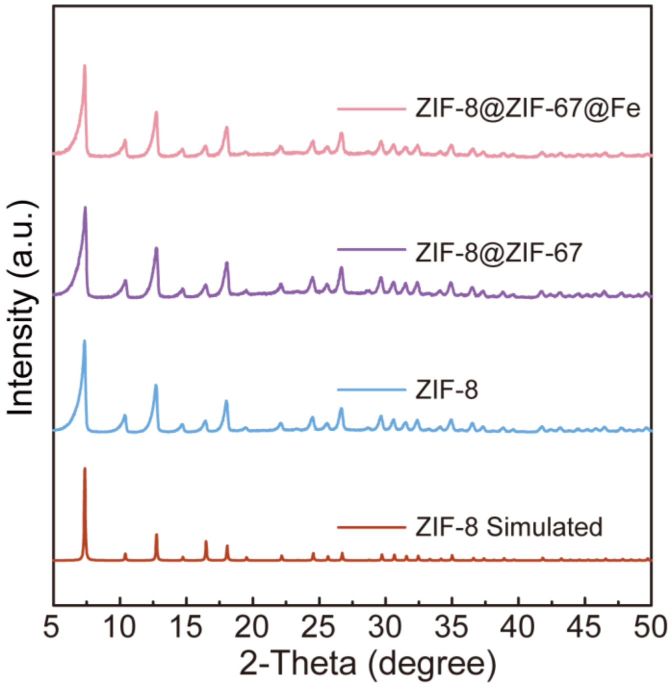

Supplementary Figure 2. XRD patterns of ZIF-8, ZIF-8@ZIF-67, and ZIF-8@ZIF-67@Fe.

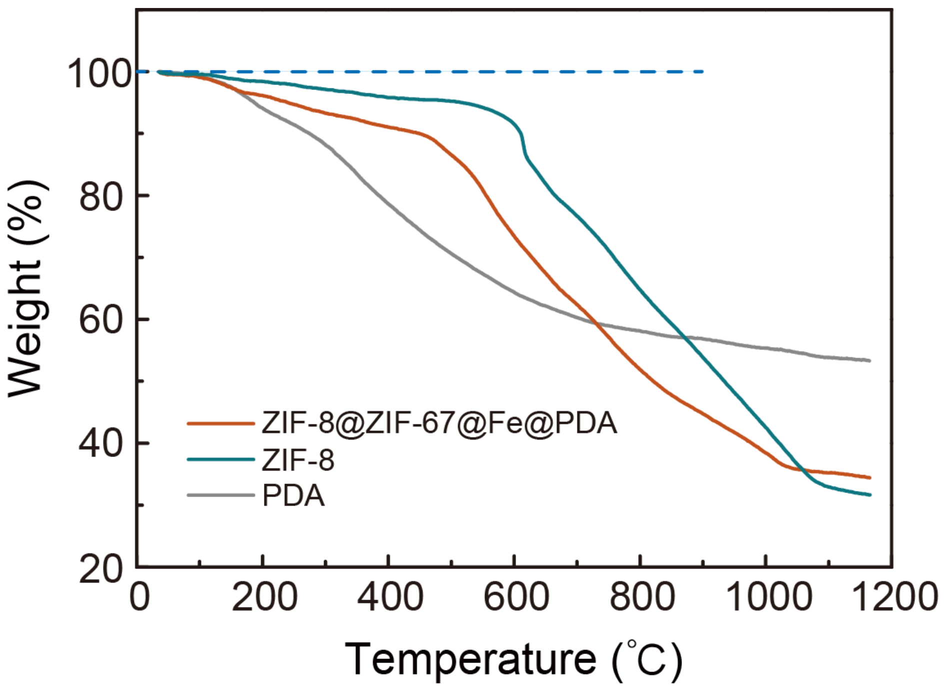

Supplementary Figure 3. TGA plots for ZIF8, PDA, and ZIF-8@ZIF-67@Fe@PDA composites. The tests were conducted in an N_2 atmosphere with a heating rate of 10 °C min^{-1}.

The PDA starts to decompose at the very early stage of pyrolysis and formed a relatively rigid carbon structure at the initial stage. The decomposition of the inner ZIF component begins at a temperature over 440 ºC. Given the interaction between the partly carbonized PDA shell and the ZIF-8 phase, the outer relatively rigid carbon shell restrains the contraction of the inner ZIF component during the pyrolysis. As the decomposition of the ZIF component goes with the temperature rising, the stresses from the shell induce the accumulation of pyrolyzed species on the shell, thus resulting in the hollow structure after the pyrolysis.

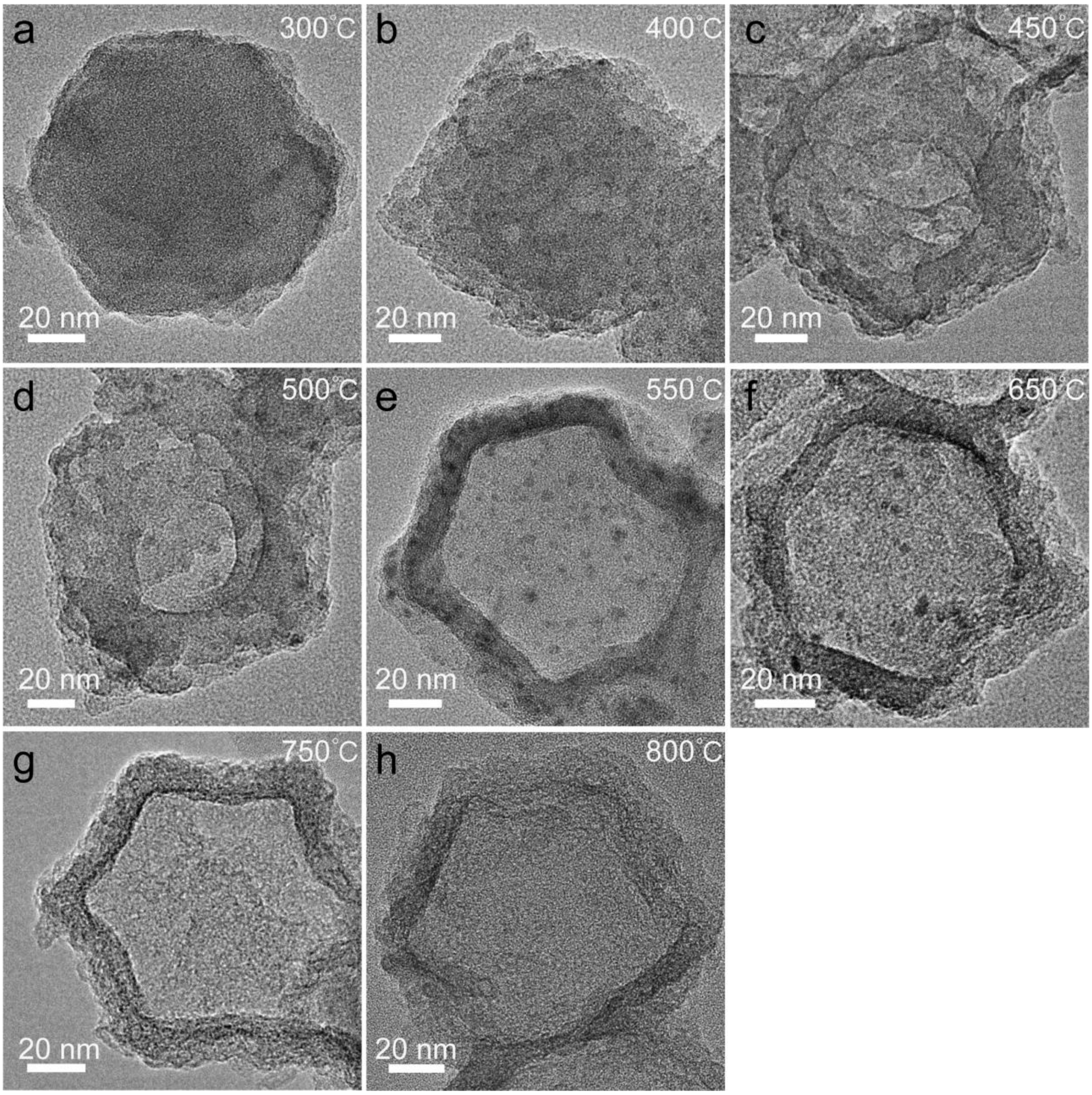

Supplementary Figure 4. TEM images collected at various temperatures during the pyrolysis for the preparation of FeCo-NCH. (a) 300 ^{o}C, (b) 400 ^{o}C, (c) 450 ^{o}C, (d) 500 ^{o}C, (e) 550 ^{o}C, (f) 650 ^{o}C, (g) 750 ^{o}C, and (h) 800 ^{o}C.

The morphology evolution of the FeCo-NCH during the pyrolysis was investigated in detail. It is found the composite surface becomes rougher but the inner remains in a solid structure at the low temperature of 300 ^{o}C, consistent with the TGA results that PDA decomposes before the ZIF component. As the temperature goes up to 400 ^{o}C at which ZIF starts to decompose, a small hollow appears, which grows as the temperature rises to 450 and 500 ^{o}C. The hollow structure formed at the temperature of 550 ^{o}C. The dark spots can be attributed to Zn-containing species from ZIF-8. As the temperature keeps rising, the carbon shell becomes thinner and the Zn species evaporate from the product. The final carbonized hollow structure was maintained at 800 ^{o}C.

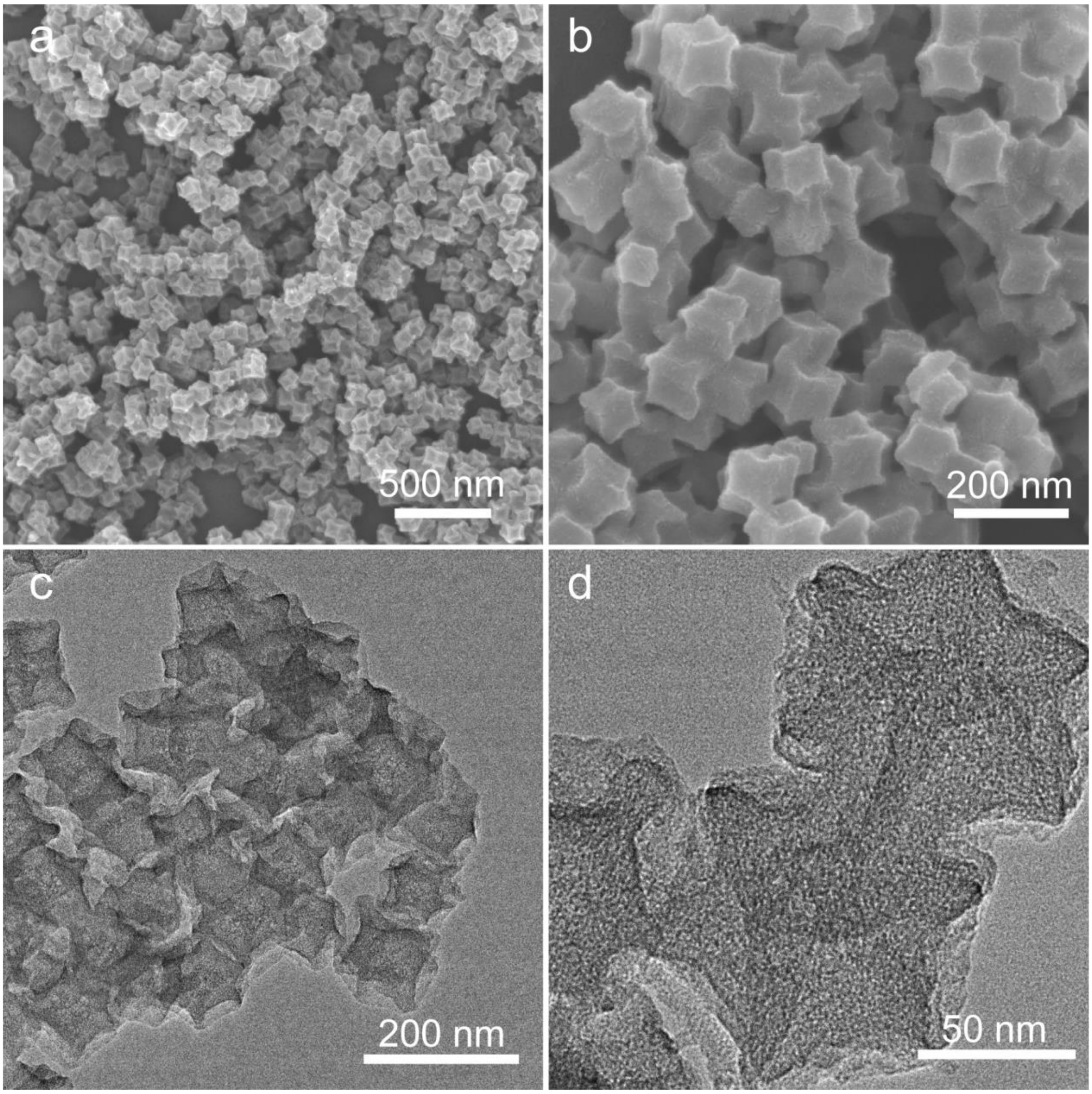

Supplementary Figure 5. (a-b) SEM and (c-d) TEM images of the control sample FeCo-NC.

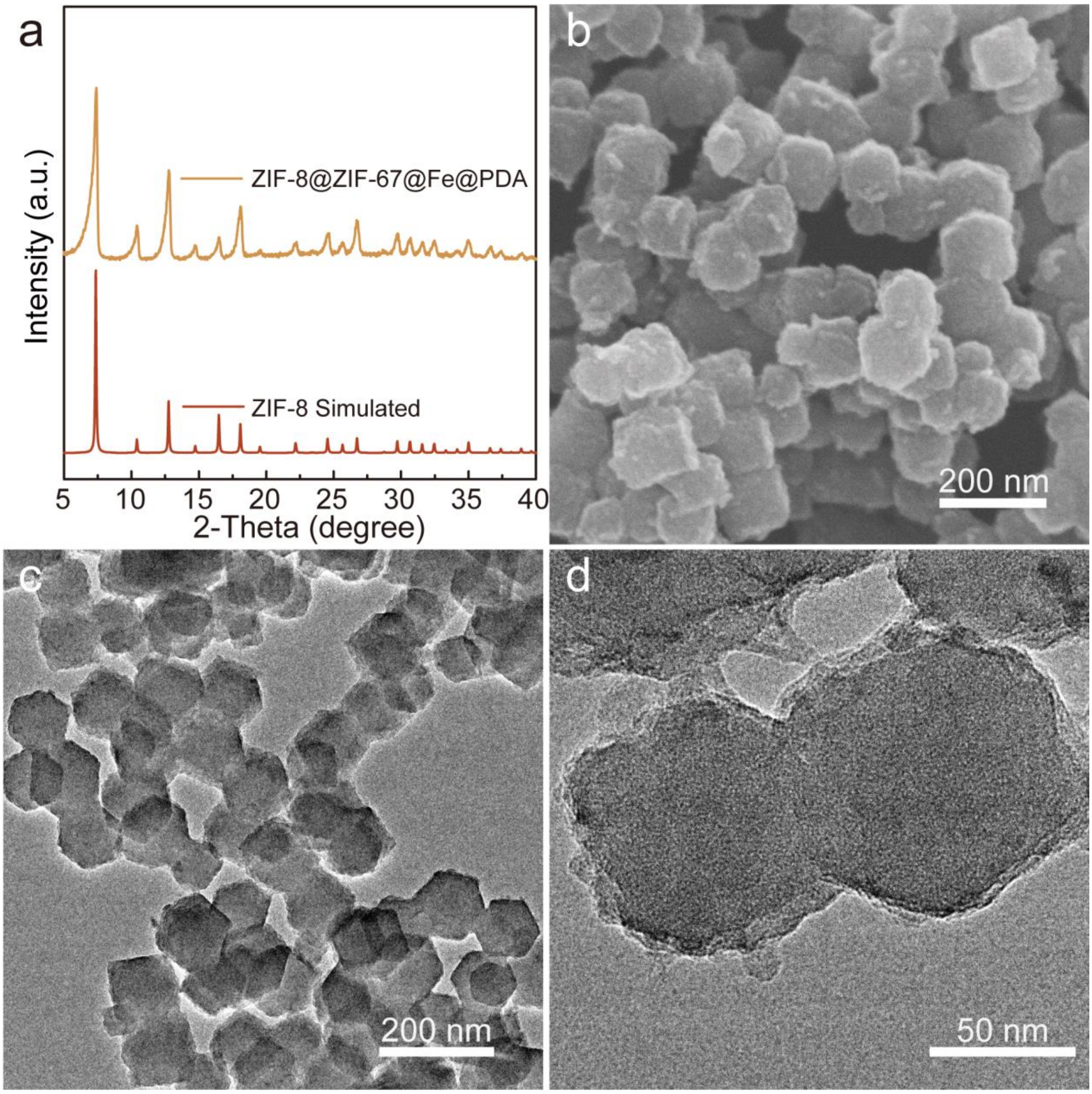

Supplementary Figure 6. (a) XRD patterns of ZIF-8@ZIF-67@Fe@PDA and simulated ZIF-8. (b) SEM, (c) TEM, and (d) HRTEM images of ZIF-8@ZIF-67@Fe@PDA.

The rough surface in the SEM image and a thin amorphous layer in the TEM and HRTEM images evidenced that ZIF-8@ZIF-67@Fe was coated with an ultra-thin shell of polydopamine (PDA).

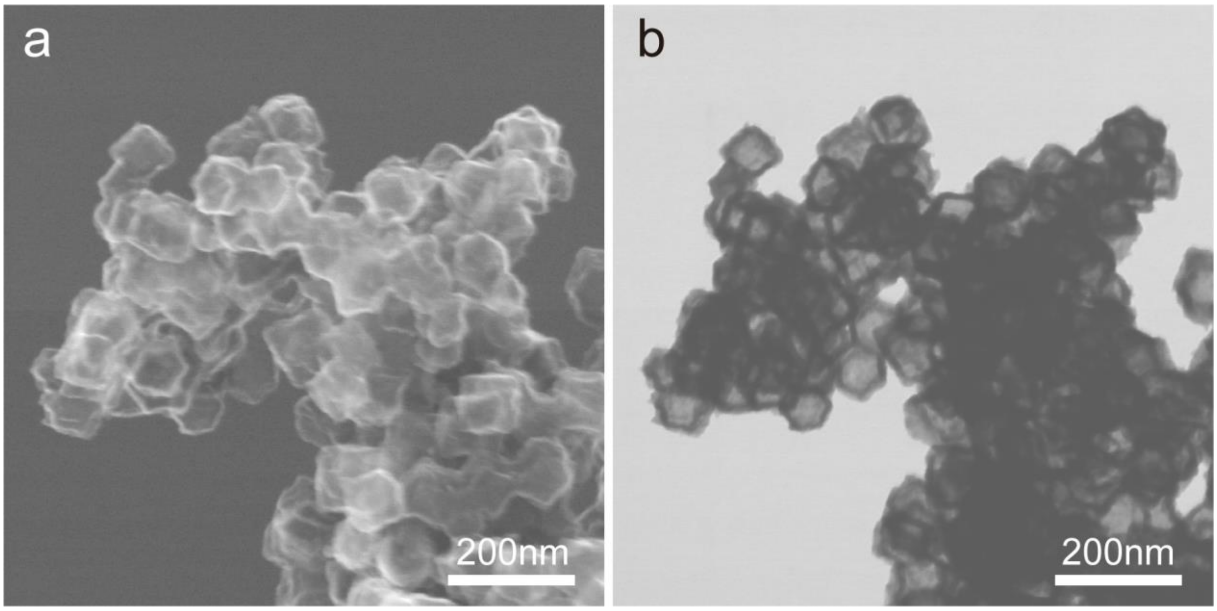

Supplementary Figure 7. (a) SEM image and (b) corresponding TEM image of FeCo-NCH.

The SEM and TEM images of FeCo-NCH captured at the same position further confirm the hollow structure.

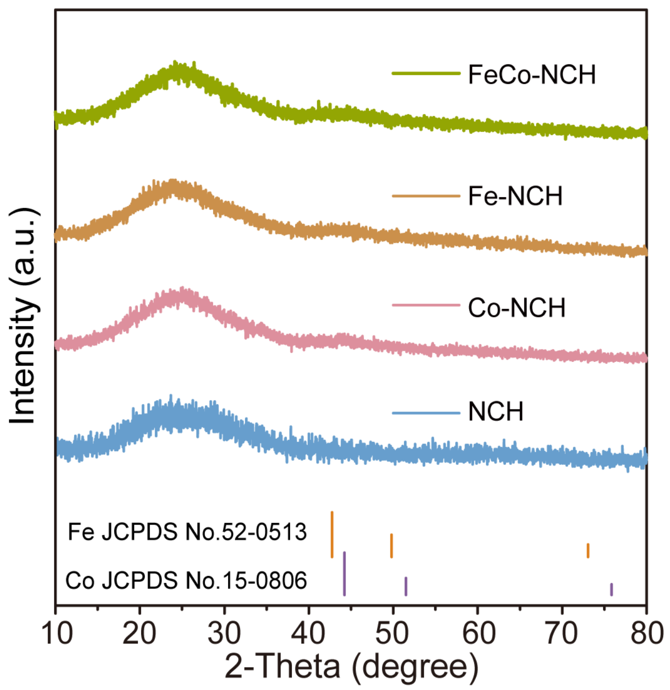

Supplementary Figure 8. XRD patterns of NCH, Co-NCH, Fe-NCH, FeCo-NCH, and the references.

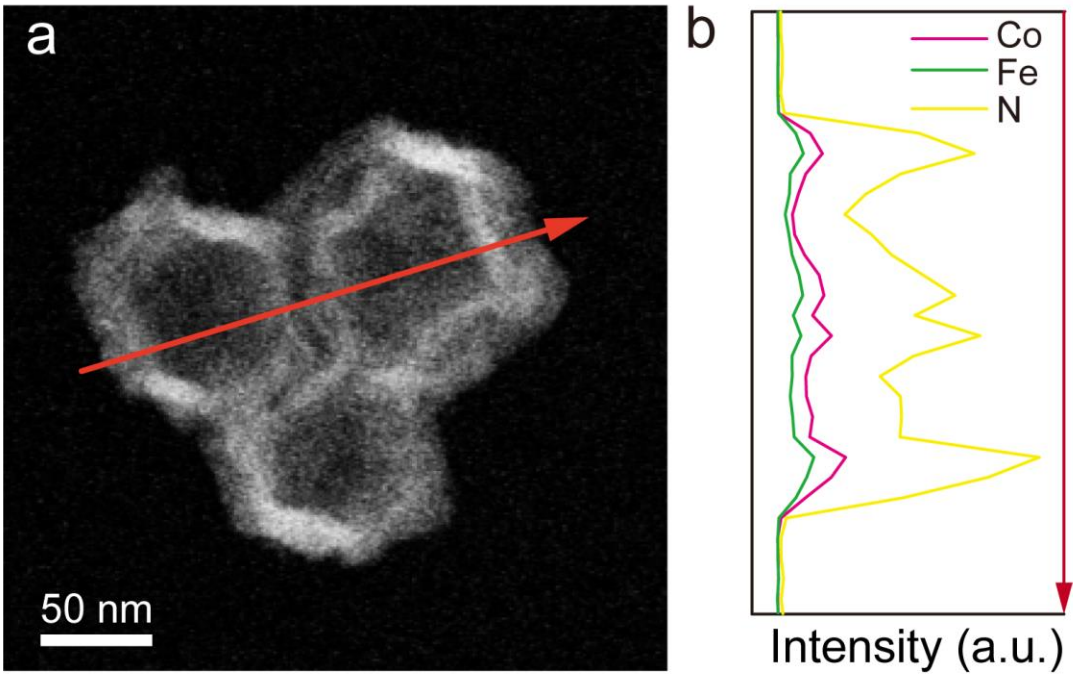

Supplementary Figure 9. (a) HAADF-STEM-EDS image, (b) corresponding linear scanning EDS analysis for FeCo-NCH.

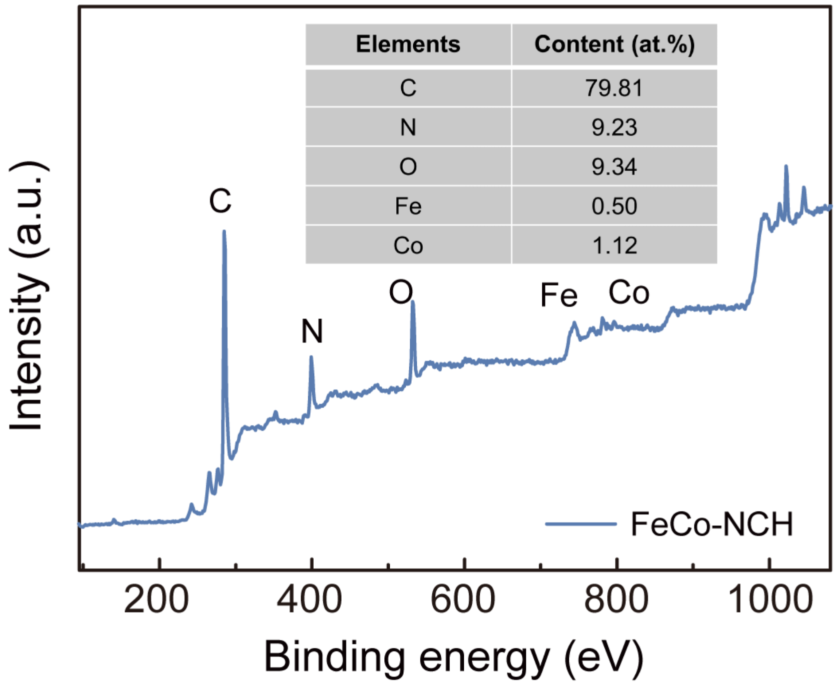

Supplementary Figure 10. The XPS survey spectra of FeCo-NCH.

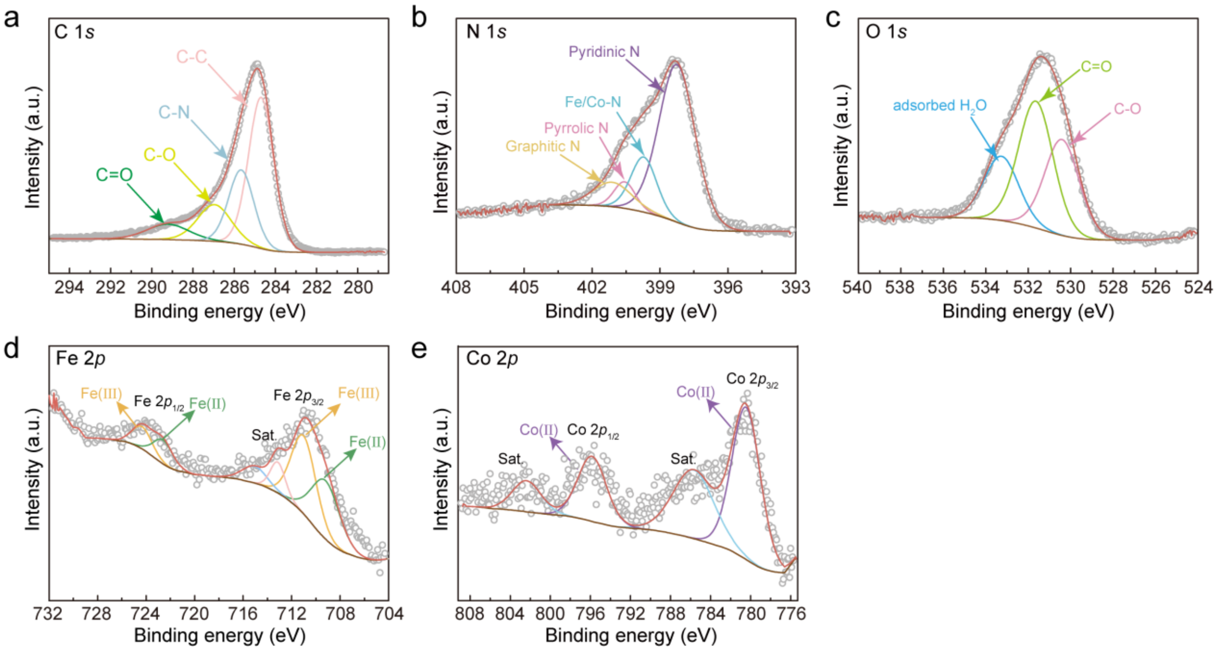

Supplementary Figure 11. XPS analysis of FeCo-NCH. (a) C 1*s* spectrum, (b) N 1*s* spectrum, (c) O 1*s* spectrum, (d) Fe 2*p* spectrum, and (e) Co 2*p* spectrum.

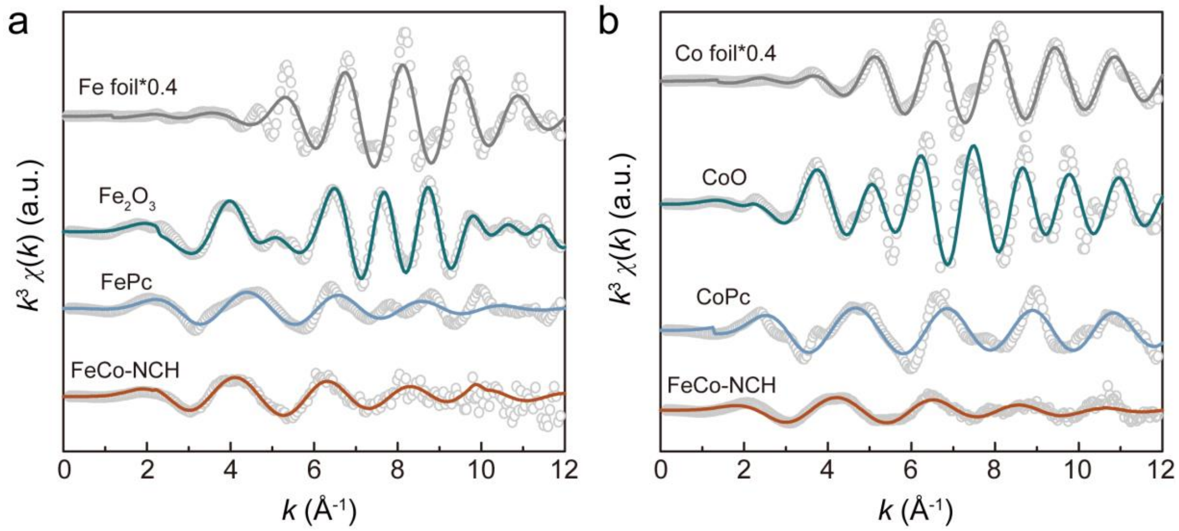

Supplementary Figure 12. (a) Fitting curves of the k^3-weighted Fe K-edge EXAFS for FeCo-NCH and reference samples. (b) Fitting curves of the k^3-weighted Co K-edge EXAFS for FeCo-NCH and reference samples.

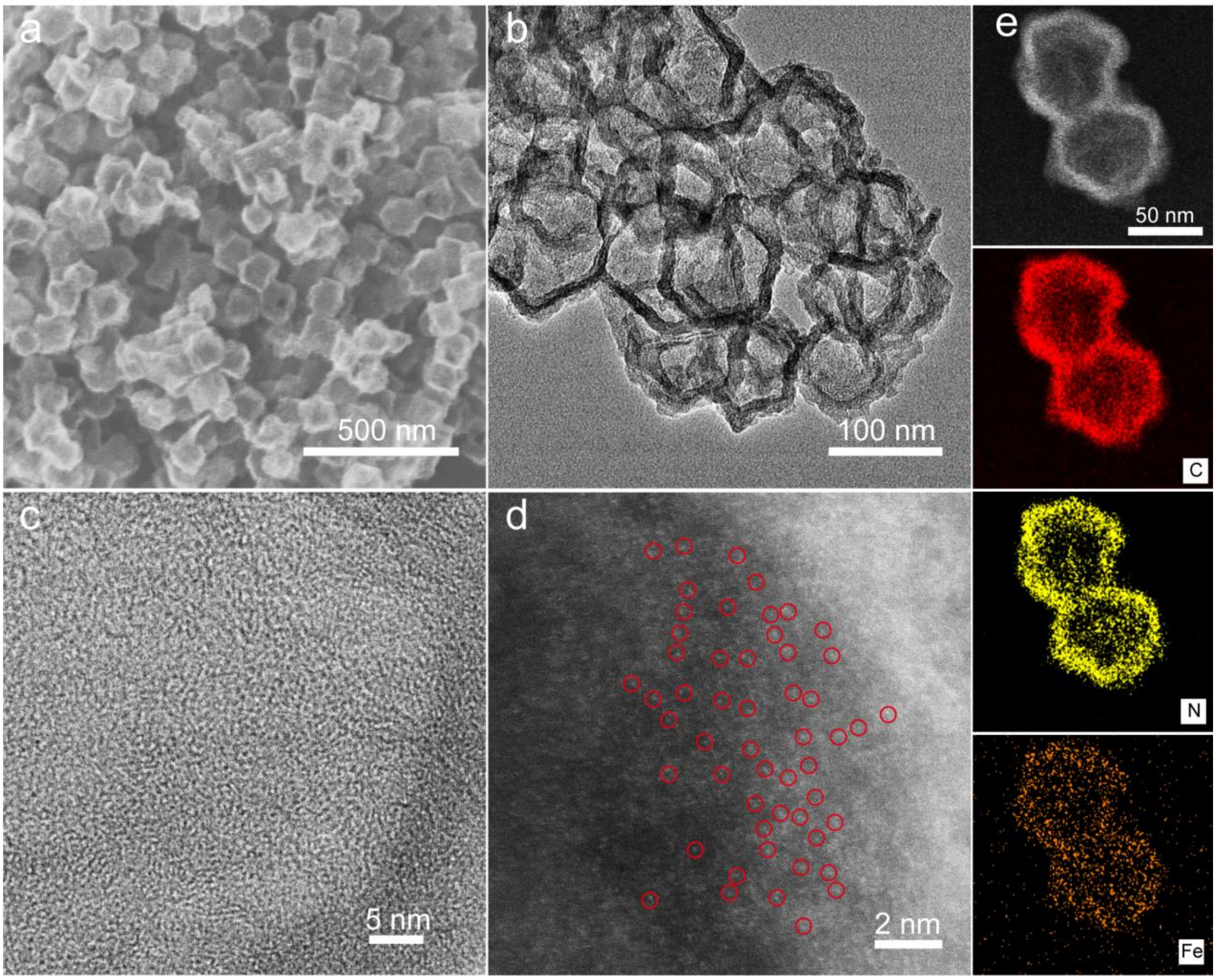

Supplementary Figure 13. (a) SEM image, (b) TEM image, (c) HRTEM image, (d) HAADF-STEM image, and (e) elemental mappings of the Fe-NCH.

The SEM, and TEM images showed that Fe-NCH shared a similar morphology to FeCo-NCH. The HRTEM image suggested that Fe existed in a single atomic state. The EDS mapping results demonstrated the uniform distribution of each element.

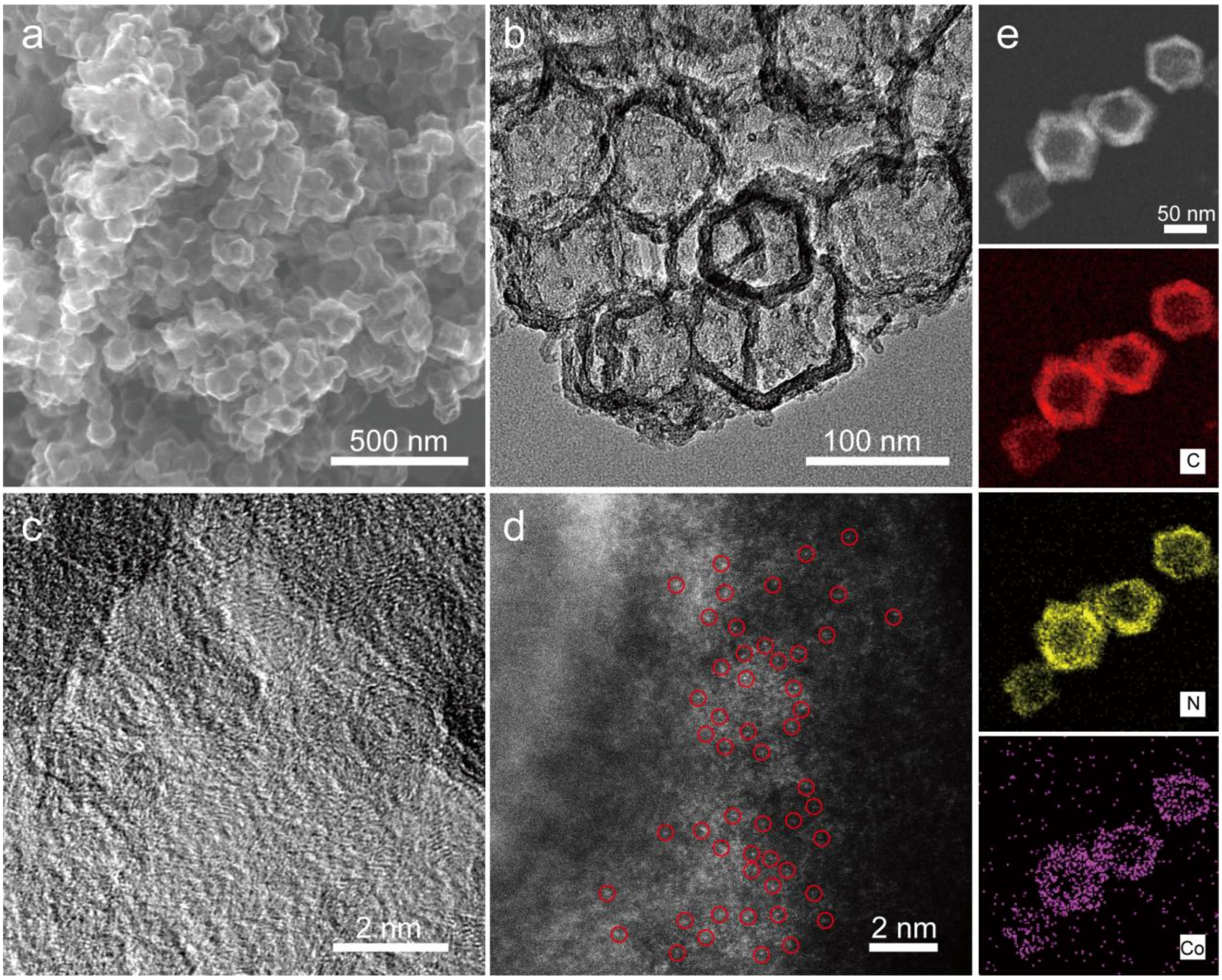

Supplementary Figure 14. (a) SEM image, (b) TEM image, (c) HRTEM image, (d) HAADF-STEM image, and (e) elemental mappings of the Co-NCH.

The SEM, and TEM images showed that Co-NCH shared a similar morphology to FeCo-NCH. The HRTEM image suggested that Co existed in a single atomic state. The EDS mapping results demonstrated the uniform distribution of each element.

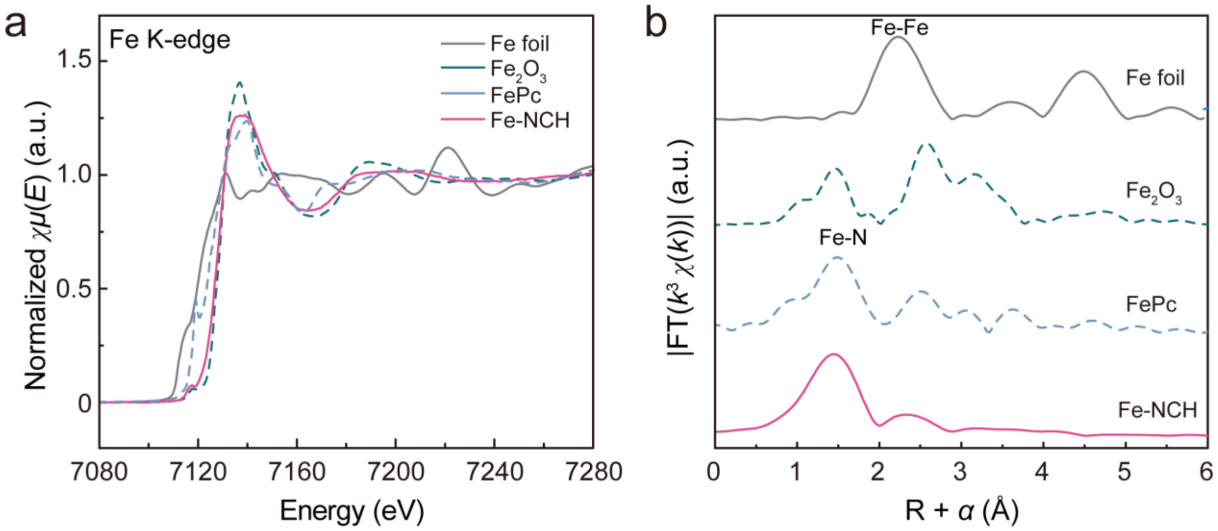

Supplementary Figure 15. (a) Fe K-edge XANES spectra of Fe-NCH in comparison with Fe foil, Fe_2O_3, and FePc. (b) Fe K-edge k^3-weighted FT-EXAFS spectra of Fe-NCH in comparison with Fe foil, Fe_2O_3, and FePc.

The Fe K-edge XANES and k^3-weighted FT-EXAFS spectra supported the existence of Fe-N_x sites in Fe-NCH.

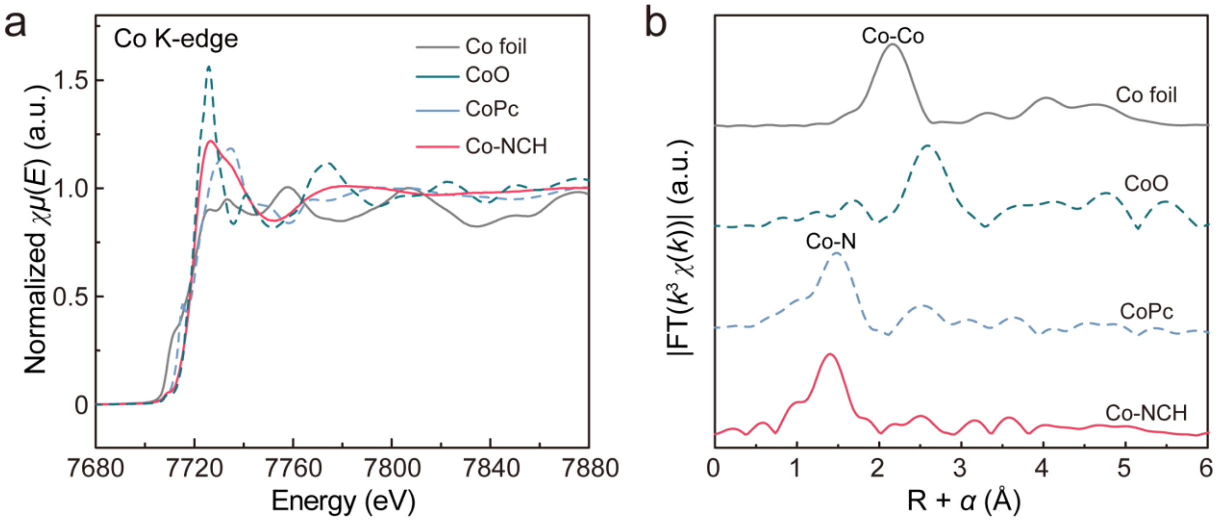

Supplementary Figure 16. (a) Co K-edge XANES spectra of Co-NCH in comparison with Co foil, CoO, and CoPc. (b) Co K-edge k^3-weighted FT-EXAFS spectra of Co-NCH in comparison with Co foil, CoO, and CoPc.

The Co K-edge XANES and k^3-weighted FT-EXAFS spectra supported the existence of Co-N_x sites in Co-NCH.

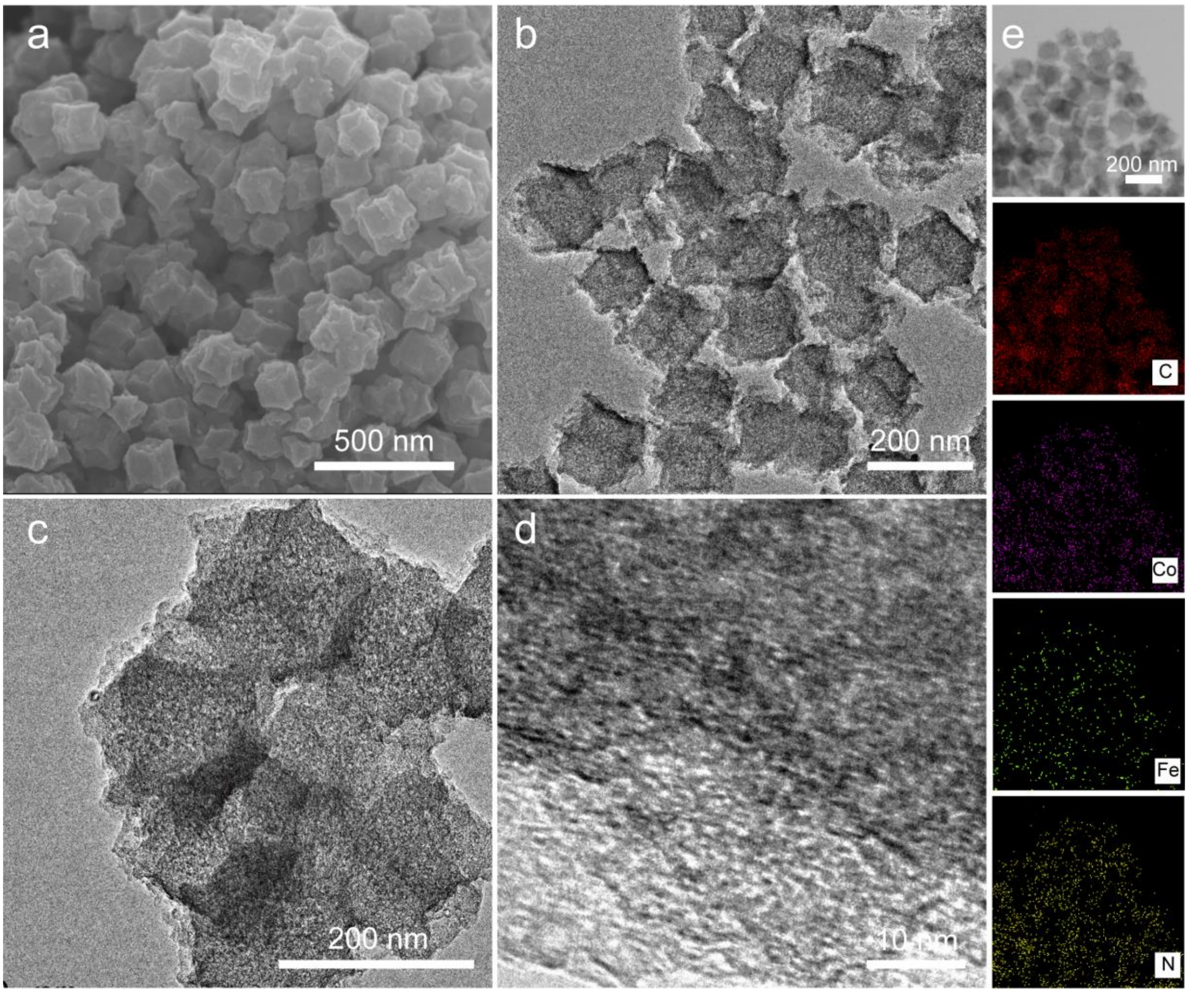

Supplementary Figure 17. (a) SEM image, (b) TEM image, (c-d) HRTEM images, and (e) elemental mappings of the FeCo-NC sample.

SEM, TEM, HRTEM, and EDS mapping results suggested that the control FeCo-NC held a similar morphology to FeCo-NCH but had a solid interior rather than the hollow one.

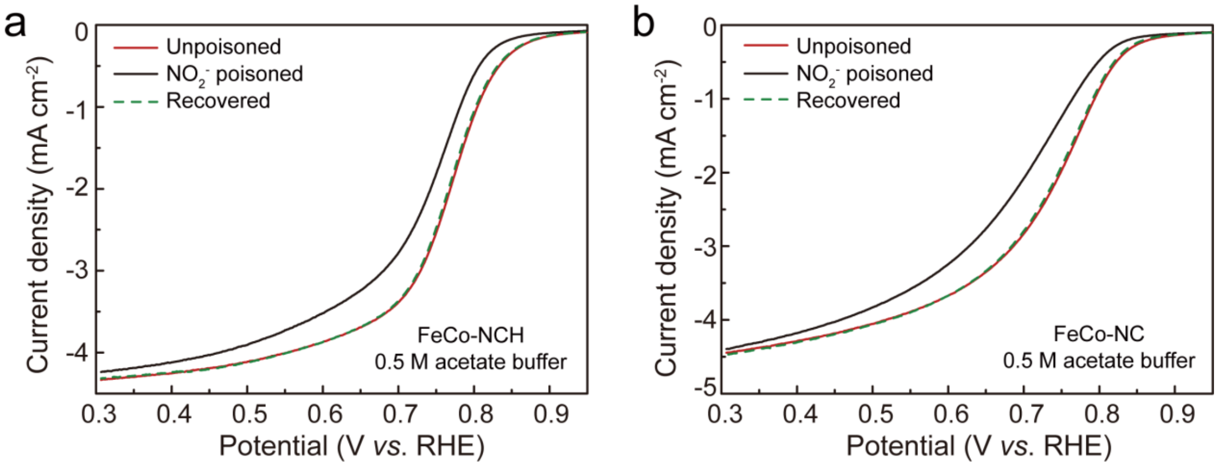

Supplementary Figure 18. LSV scanning experiments to determine the SD of (a) FeCo-NCH and (b) FeCo-NC through reversible nitrite poisoning in O_2-saturated 0.5 M acetate buffer under a rotating rate of 1600 rpm.

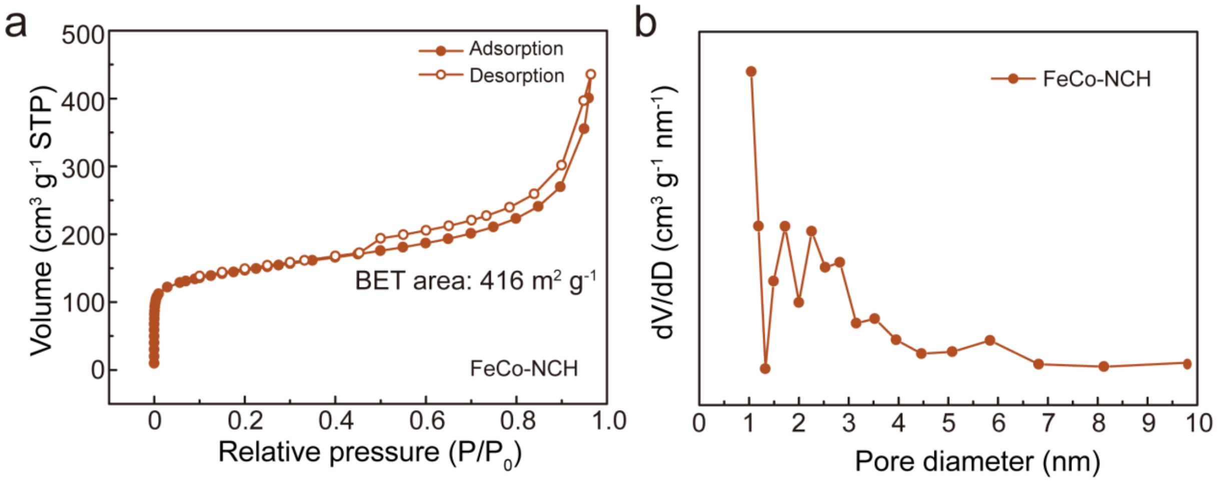

Supplementary Figure 19. (a) N_2 adsorption-desorption isotherms and (b) pore size distribution of the FeCo-NCH.

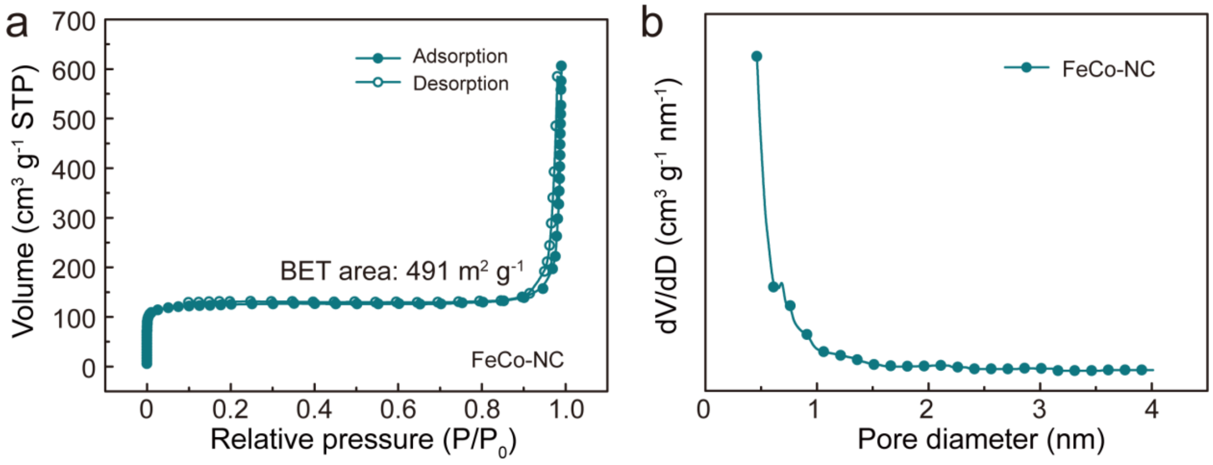

Supplementary Figure 20. (a) N_2 adsorption-desorption isotherms and (b) pore size distribution of the FeCo-NC.

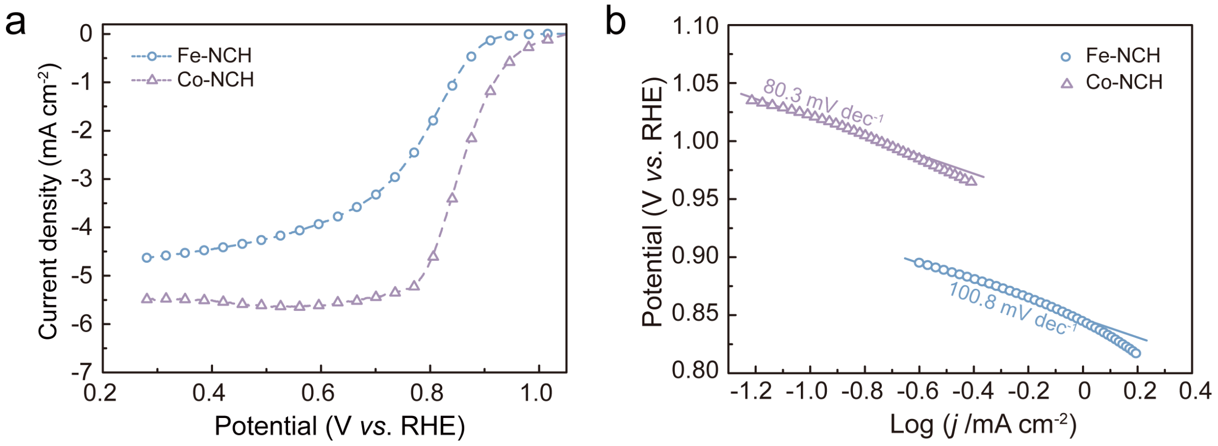

Supplementary Figure 21. (a) Steady-state ORR polarization curves of Co-NCH and Fe-NCH in O_2-saturated 0.1 M KOH under a rotating rate of 1600 rpm. (b) The corresponding Tafel plots for Co-NCH and Fe-NCH.

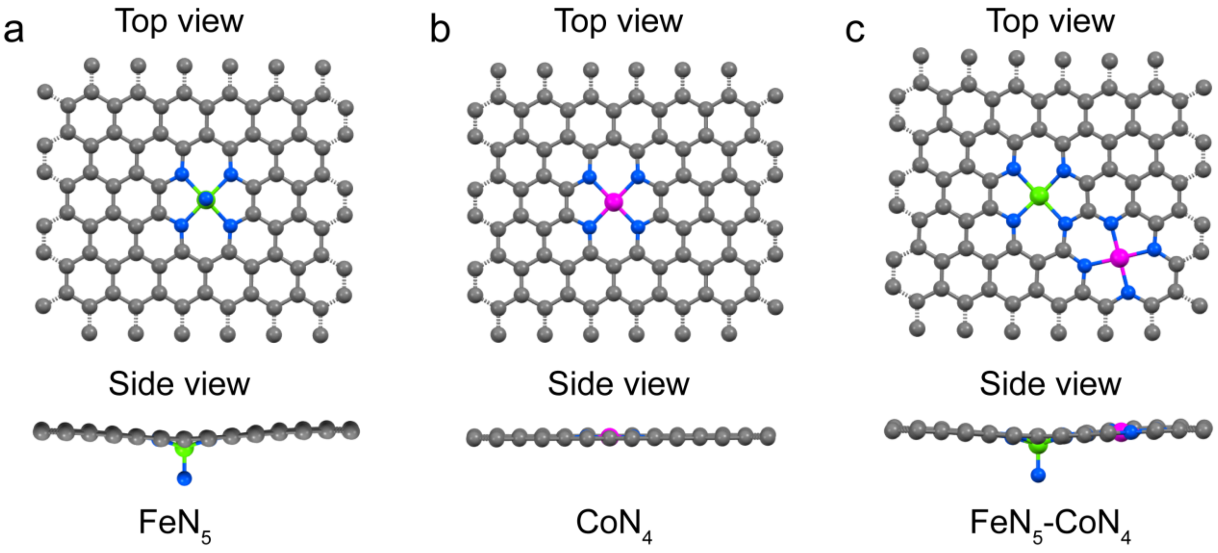

Supplementary Figure 22. Illustrations of optimized atomic configurations of (a) FeN_5, (b) CoN_4, and (c) neighboring FeN_5-CoN_4 model. (C: gray, N: blue, Fe: green, Co: pink, O: red, H: white).

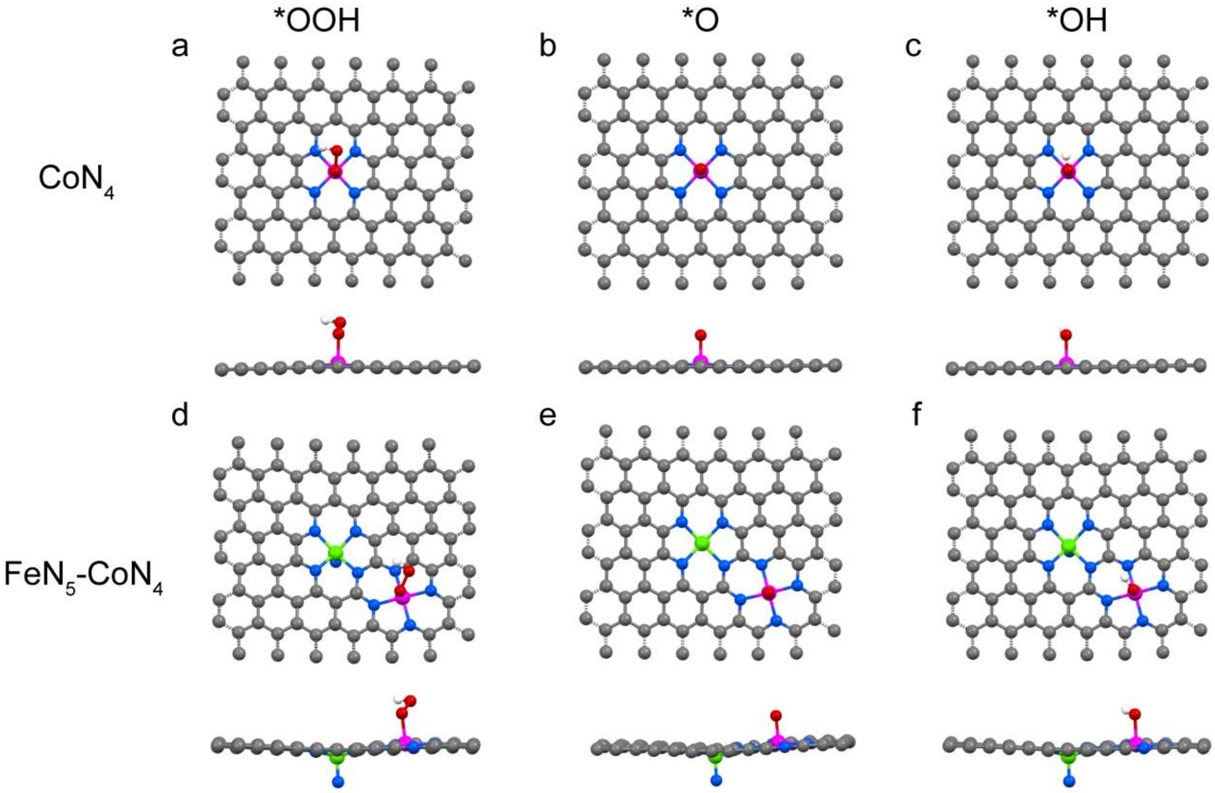

Supplementary Figure 23. (a-c) Configurations of corresponding adsorbed intermediates (*OOH, *O, and *OH) on CoN_4 and (d-f) FeN_5-CoN_4 models (C: gray, N: blue, Fe: green, Co: pink, O: red, H: white).

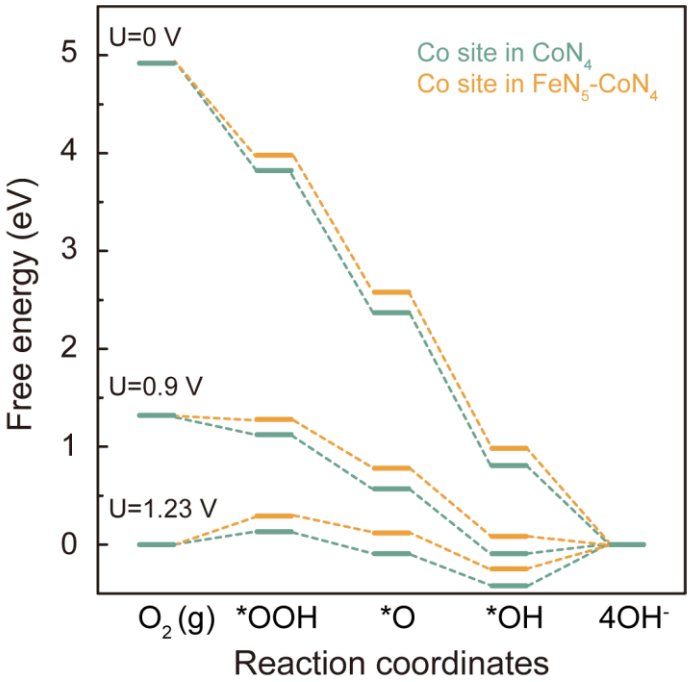

Supplementary Figure 24. ORR free energy diagrams for the Co site in Co-N_4 (green line) and in FeN_5-CoN_4 models (orange line) at U=0 V, 0.9 V, and 1.23 V.

For Co sites, all the elementary reaction steps on both FeN_5-CoN_4 and CoN_4 models present a consistent downhill tendency at the U = 0 V, implying a spontaneous exothermal process. Upon increasing the thermodynamic equilibrium potential to 0.9 V, the ORR process on the FeN_5-CoN_4 model is still spontaneous, while the CoN_4 possesses a slight endothermic process from the desorption of *OH in the fourth step, suggesting that the external force is needed to drive this process. At U = 1.23 V, the rate-determining step (RDS) in the CoN_4 model is the desorption of adsorbed *OH with the largest Gibbs free energy change ΔG_4 of 0.42 eV. In contrast, the corresponding ΔG_4 in the FeN_5-CoN_4 model is much small (0.25 eV). The RDS for the FeN_5-CoN_4 model is the adsorption of the *OOH from the first electron transfer step with a ΔG_1 of 0.29 eV. The limiting reaction energy barrier of ORR on FeN_5-CoN_4 (0.29 eV) is lower compared with the CoN_4 model (0.42 eV). These results indicate that the introduction of the Fe site promotes the desorption of *OH on the Co site and optimizes the ORR pathway.

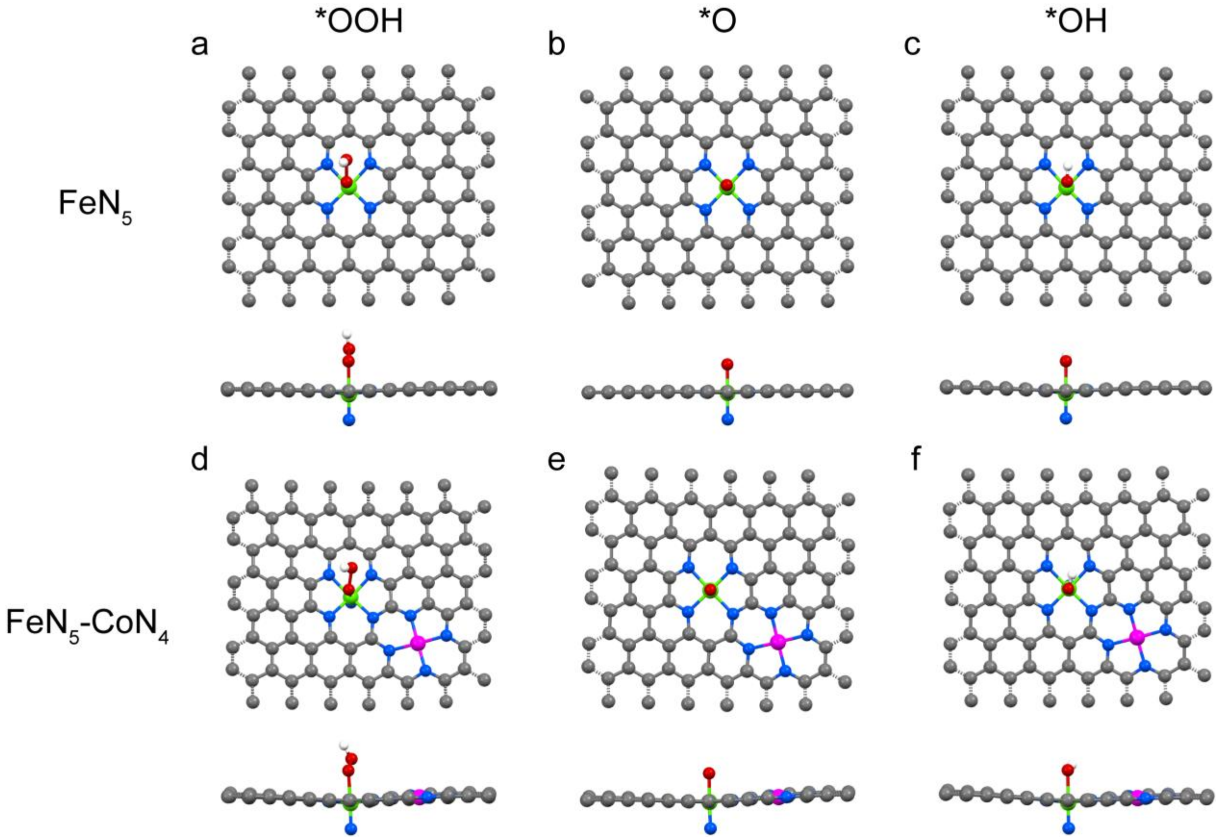

Supplementary Figure 25. (a-c) Configurations of corresponding adsorbed intermediates (*OOH, *O, and *OH) on FeN_5 and (d-f) FeN_5-CoN_4 models (C: gray, N: blue, Fe: green, Co: pink, O: red, H: white).

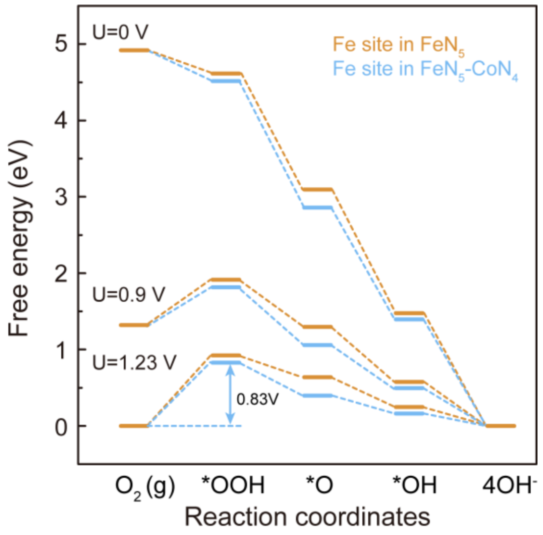

Supplementary Figure 26. ORR free energy diagrams for the Fe site in FeN_5 (orange line) and in FeN_5-CoN_4 models (blue line) at U=0 V, 0.9 V, and 1.23 V.

As for Fe sites, when U = 0 V, all the elementary reaction steps on FeN_5-CoN_4 and FeN_5 models present a consistent downhill tendency, implying a spontaneous exothermal process. Upon increasing the thermodynamic equilibrium potential to 0.9 V, both the FeN_5-CoN_4 and FeN_5 present the uphill free energy in the first electron transfer step ($O_2 + H_2O + e^- \rightarrow$ *OOH + OH^-), which is the RDS for the two models. It is noted that the FeN_5-CoN_4 model gives a lower energy barrier of 0.49 eV than that of the FeN_5 model (0.59 eV). Upon increasing the potential to 1.23 V, the first electron transfer step remains to be the RDS. The limiting reaction energy barrier of ORR on the FeN_5-CoN_4 (0.83 eV) model is still lower than that on the FeN_5 (0.92 eV) model, implying the ORR on the Fe site in FeN_5-CoN_4 is energetically favorable to that in the FeN_5. These results suggest that the introduction of the Co site significantly reduces the *OOH formation energy barrier on the Fe site and optimizes the ORR pathway.

Notably, the higher ORR performance of Co-NCH than Fe-NCH can be attributed to the following two reasons.

(1) Higher intrinsic activity of Co-N_4 compared to the Fe-N_5 sites. The DFT calculation results show that the ORR process is more favorable on the Co-N_4 sites than that on the Fe-N_5 sites. The RDS of the Co-N_4 model is the last electron transfer step of the desorption of *OH with a free energy of 0.42 eV. While for the FeN_5 model, the RDS is *OOH adsorption with a free energy of 0.92 eV, which is much higher than that of the CoN_4 model. This result implies that CoN_4 may possess a better ORR activity than FeN_5 in our case.

(2) Co-NCH has more active sites than Fe-NCH. The metal loading of Co-NCH is 5.5 wt% vs. 2.4 wt% of Fe in Fe-NCH. The higher metal loading corresponds to more functional sites to catalyze the ORR. As shown in Supplementary Fig. 21, the Co-NCH exhibits a larger limiting current density than the Fe-NCH, indicating that more active sites participate in the ORR process than the Fe-NCH.

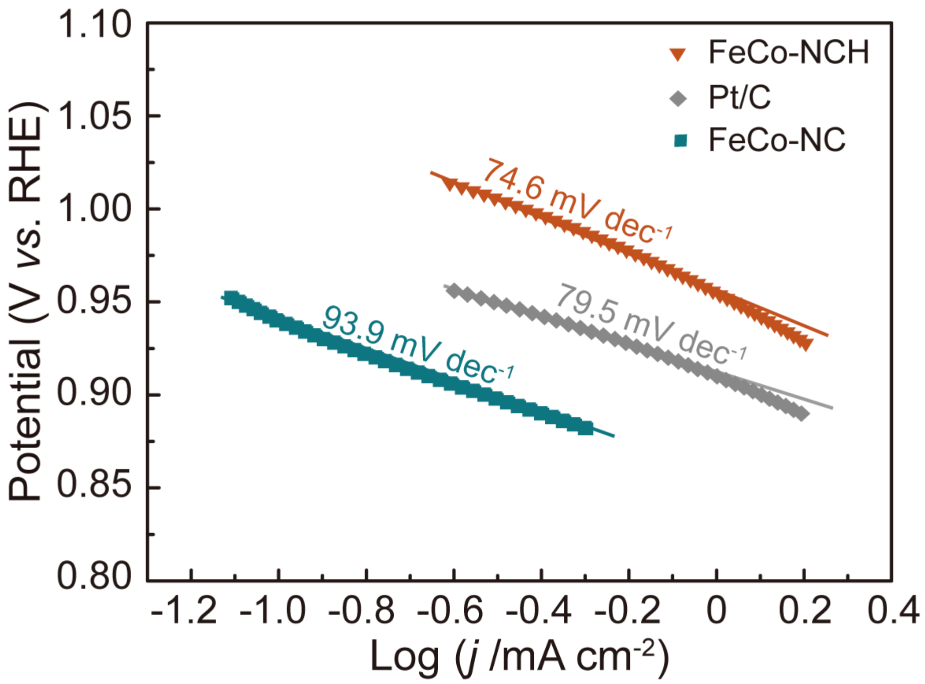

Supplementary Figure 27. The corresponding Tafel plots for FeCo-NCH, Pt/C, and FeCo-NC.

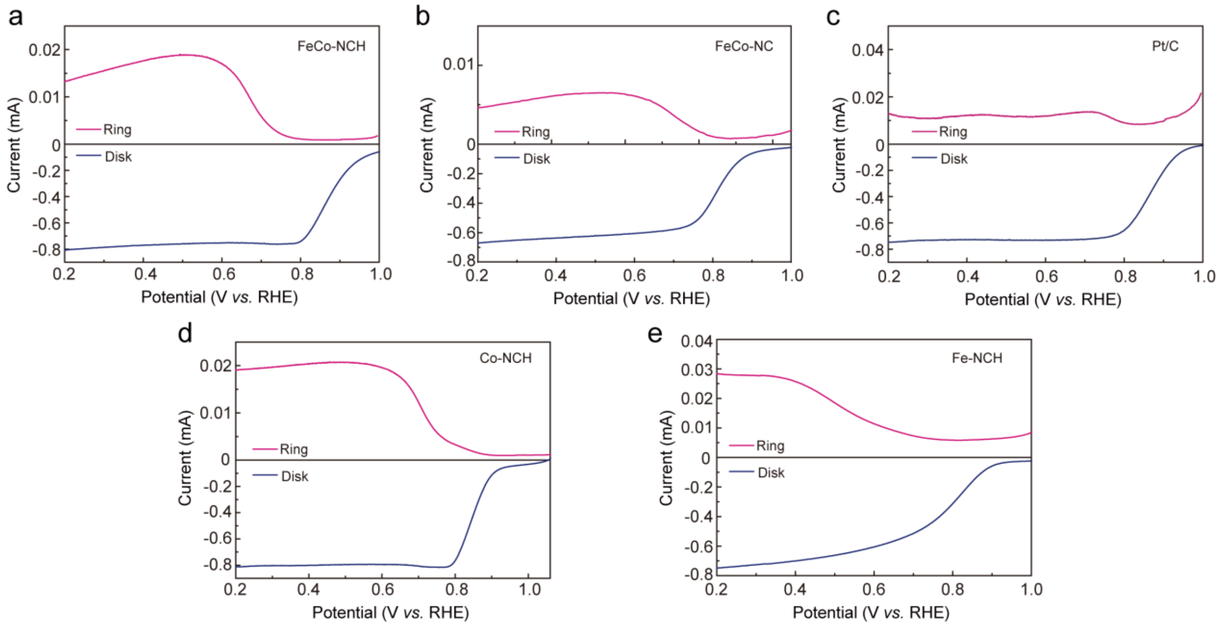

Supplementary Figure 28. RRDE measurement curves of (a) FeCo-NCH, (b) FeCo-NC, (c) Pt/C, (d) Co-NCH, and (e) Fe-NCH, recorded in O_2-saturated 0.1 M KOH under a rotating rate of 1600 rpm.

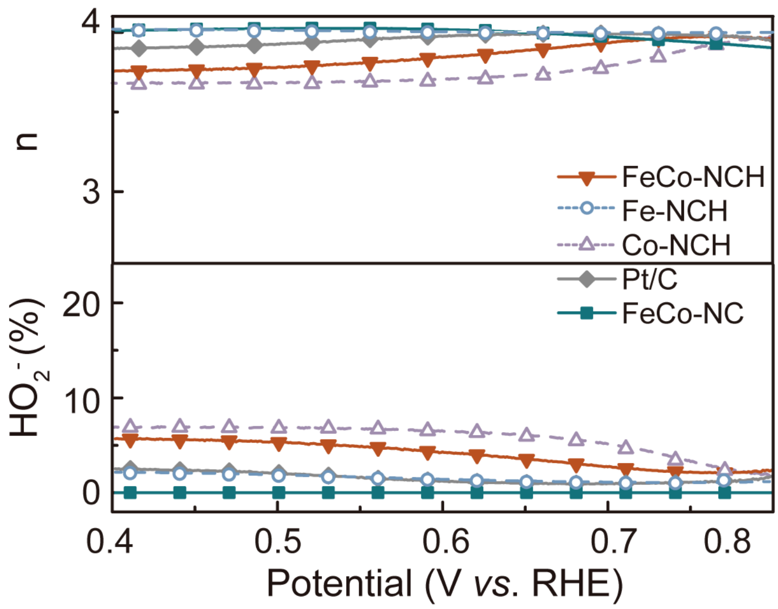

Supplementary Figure 29. HO_2^- yield and calculated electron transfer number during ORR for FeCo-NCH, Pt/C, and control samples.

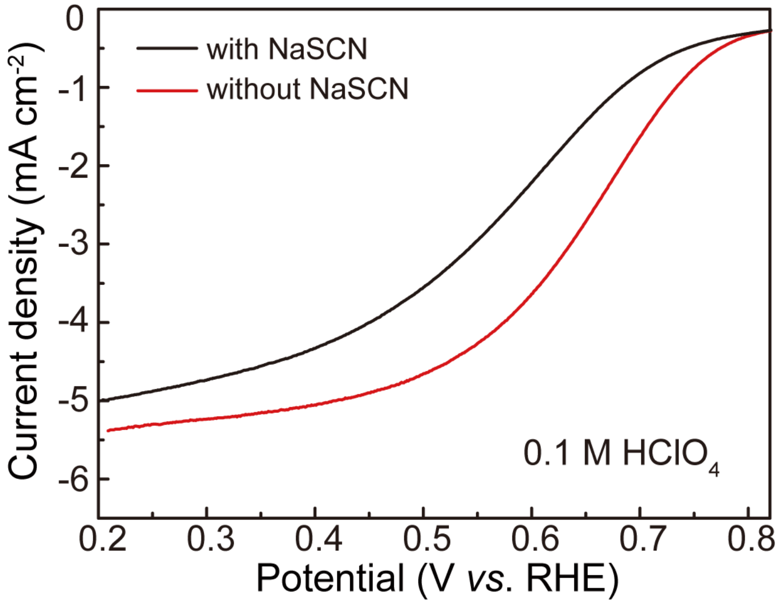

Supplementary Figure 30. Poisoning experiments of FeCo-NCH in an O_2-saturated 0.1 M $HClO_4$ electrolyte under a rotating rate of 1600 rpm with and without NaSCN in the electrolyte. The significantly degraded ORR activity suggested that the active sites should be ascribed to metal-N_x sites.

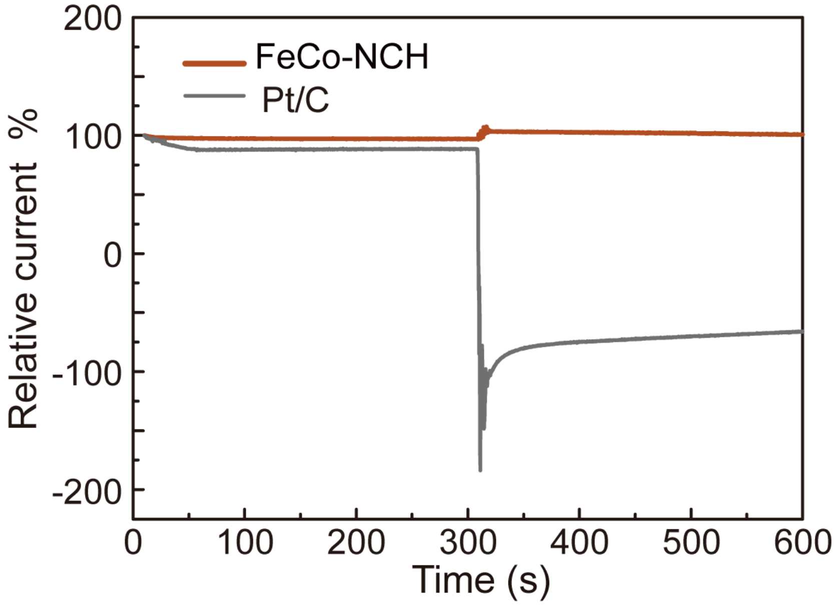

Supplementary Figure 31. Methanol tolerance tests for FeCo-NCH and commercial Pt/C with the addition of 3 M methanol.

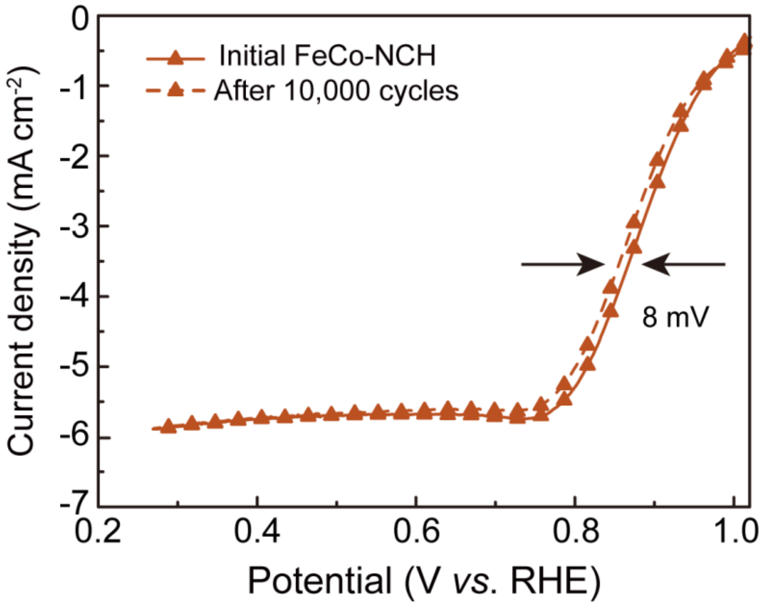

Supplementary Figure 32. ORR LSV curves of FeCo-NCH before and after 10,000 cycles between 0.6 and 1.0 V at a scan rate of 100 mV s^{-1}.

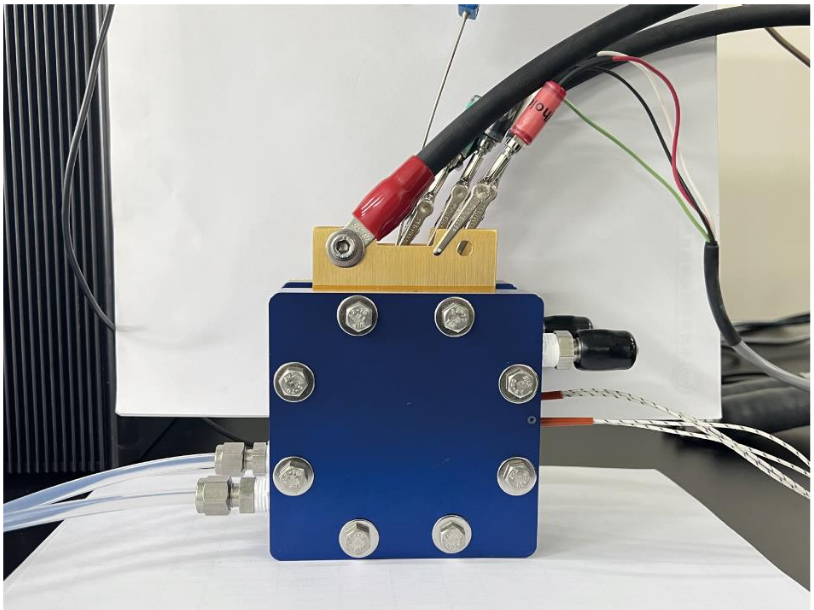

Supplementary Figure 33. Photograph of the AEMFC.

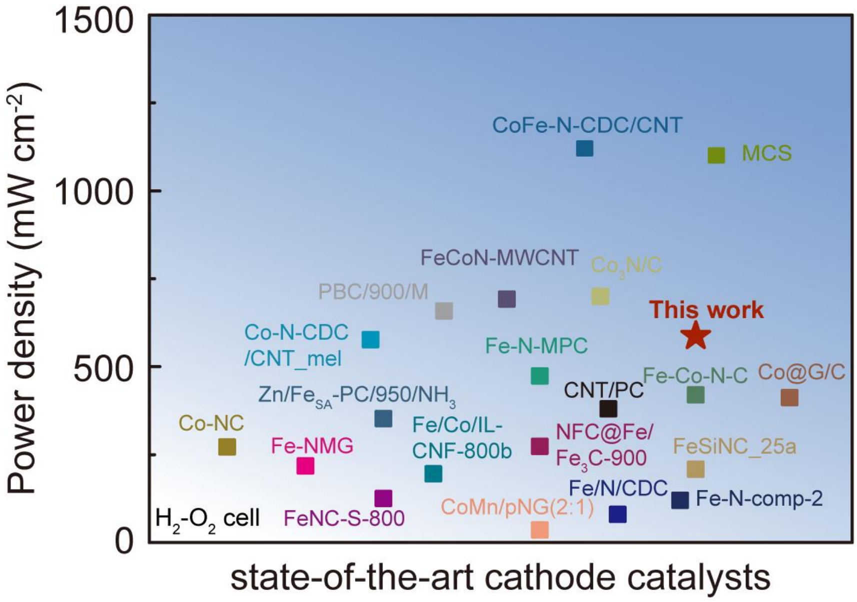

Supplementary Figure 34. Peak power density comparison of the FeCo-NCH with reported Pt-free catalysts in H_2-O_2 AEMFCs.

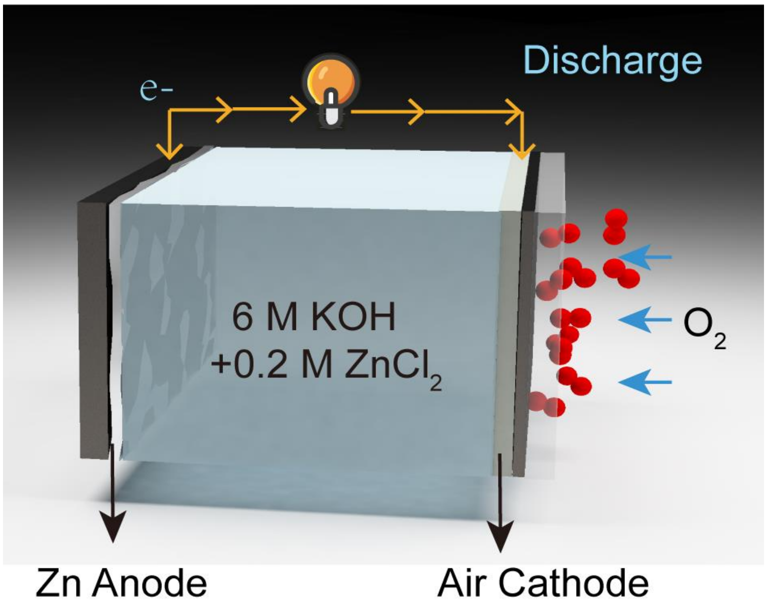

Supplementary Figure 35. Schematic illustration of the aqueous Zn-air battery.

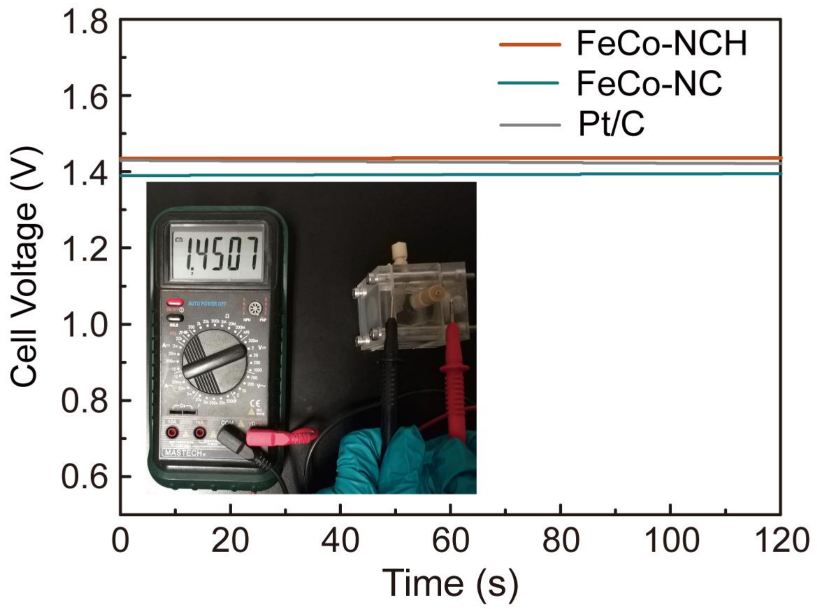

Supplementary Figure 36. Open-circuit voltages of the Zn-air battery. The inset is the photograph of the FeCo-NCH-based Zn-air battery, showing an OCV of 1.45 V.

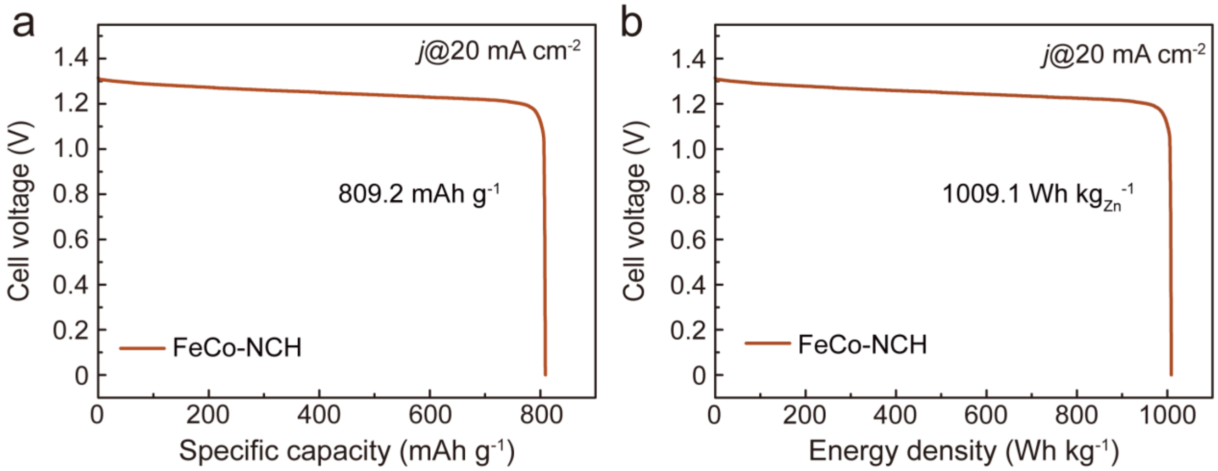

Supplementary Figure 37. Discharge (a) specific capacity and (b) energy density plots of FeCo-NCH at 20 mA cm^{-2}.

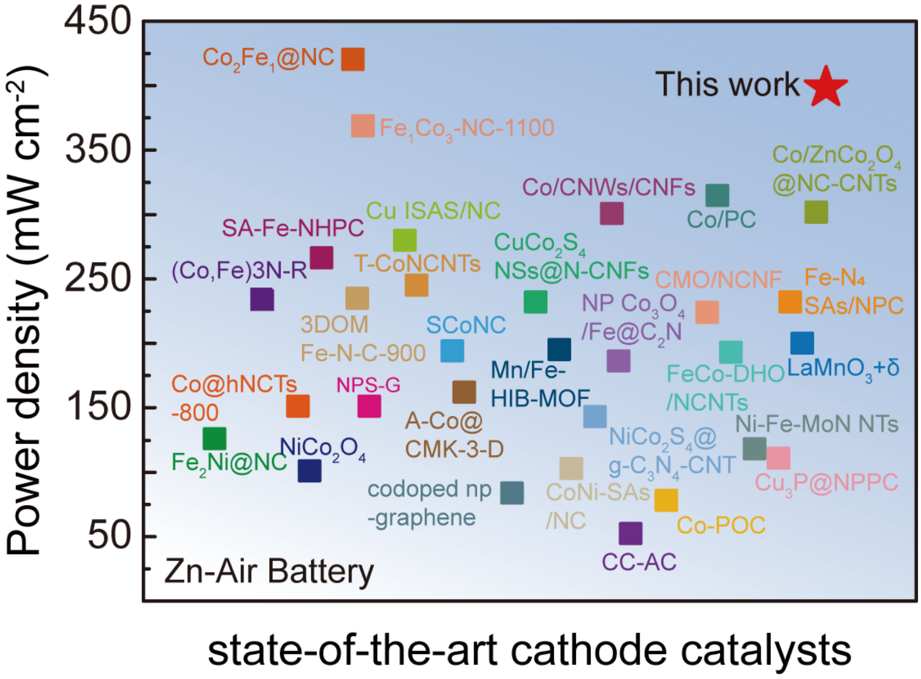

Supplementary Figure 38. Peak power density comparison of the FeCo-NCH with state-of-the-art Zn-air battery cathode catalysts.

Supplementary Tables

Supplementary Table 1. EXAFS fitting parameters at the Co, Fe K-edge for various samples (S_0^2 = 0.829 (Fe), S_0^2=0.776 (Co))

Sample	Shell	N^a	$R(Å)^b$	$\sigma^2(Å^2)^c$	ΔE_0 (eV)^d	R factor
			**Fe K-edge**			
**Fe foil**	Fe-Fe	8	2.46	0.0045	5.0	0.0017
	Fe-Fe	6	2.85	0.0045		
**Fe_2O_3**	Fe-O	6.0	2.04	0.0095	0.1	0.0008
	Fe-Fe	6.0	2.97	0.0075		
	Fe-Fe	4.1	3.39	0.0073		
	Fe-Fe	5.7	3.70	0.0073		
**FePc**	Fe-N	4.0	1.96	0.0035	1.4	0.0014
**FeCo-NCH**	Fe-N	4.9	2.01	0.0080	-2.8	0.0004
			**Co K-edge**			
**Co foil**	Co-Co	12	2.49	0.0062	7.1	0.0004
**CoO**	Co-O	6.0	2.06	0.0111	-5.6	0.0003
	Co-Co	12.5	2.99	0.0091		
	Co-O	6.2	3.66	0.0101		
**CoPc**	Co-N	4.0	1.95	0.0015	4.2	0.0078
**FeCo-NCH**	Co-N	4.1	1.93	0.0100	-7.2	0.0007

$^a N$: coordination numbers; $^b R$: bond distance; $^c \sigma^2$: Debye-Waller factors; $^d \Delta E_0$: the inner potential correction. R factor: goodness of fit. S_0^2 was set to 0.829(Fe), 0.776(Co), according to the experimental EXAFS fit of metal foil reference by fixing CN as the known crystallographic value.

Supplementary Table 2. Metal content comparison of dual-site SACs according to the recently reported MOF-derived catalysts.

Catalysts	Content of metal elements (wt%)	Ref.
**FeCo-NCH**	**Co: 5.5 Fe: 2.4**	**This work**
(Fe,Co)/N-C	Co: 1.17 Fe: 0.93	1
FeCo-NPC	Fe: 0.33 Co: 0.14	2
Fe, Mn-N/C-900	Fe: 1.75 Mn: 0.07	3
FeCo-ISAc/NC	Co: 0.218 Fe: 0.964	4
CoFe@C	Co: 0.5 Fe: 0.37	5
Ni/Fe–N–C	Ni: 0.97 Fe: 0.34	6
Zn/CoN–C	Co: 0.37 Fe: 0.43	7
$FeCoN_5C$	Co: 1.12 Fe: 1.06	8
FeCo-IA/NC	Co: 1.06 Fe: 0.26	9
$FeNi\text{-}N_6\text{-}C$	Ni: 1.472 Fe: 1.448	10
$Co_1\text{-}PNC/Ni_1\text{-}PNC$	Co: 2.15 Ni: 0.68	11
$Fe/Ni\text{-}N_x/OC$	Fe: 1.37 Ni: 0.47	12
ZnCoNC	Zn: 0.35 Co: 0.72	13
$Co_2/Fe\text{-}N@CHC$	Co: 2.13 Fe: 0.98	14
Ni/Cu-N-C	Ni: 0.27 Cu: 0.31	15
Ni-Zn-N-C	Ni: 0.84 Zn: 1.32	16
$Fe_1\text{-}Ni_1\text{-}N\text{-}C$	Fe: 0.45 Ni: 0.42	17
NiFe-DASC	Ni: 4.05 Fe: 3.24	18
ZnLa-1/CN	Zn: 2.52 La: 1.16	19
Fe/Cu-N-C	Fe: 0.30 Cu: 0.19	20

Supplementary Table 3. Metal contents in various catalysts, obtained by XPS and ICP.

Samples		ICP (wt%)	XPS (wt%)
FeCo-NCH	Fe	2.4	2.1
	Co	5.5	4.6
FeCo-NC	Fe	2.1	0.6
	Co	5.0	2.4
Fe-NCH	Fe	2.7	2.7
Fe-NC		1.9	1.1
Co-NCH	Co	5.2	4.9
Co-NC		4.5	2.2

Supplementary Table 4. Comparison of the RDE and AEMFC performances for our electrocatalysts and various reported PGM-free cathode electrocatalysts (H_2/O_2 and H_2/Air).

**Catalysts**	**Ionomer**	**Membrane**	***$E_{1/2}$* (V *vs.* RHE)**	***P_{max}* (H_2-O_2) (mW cm^{-2})**	***P_{max}* (H_2-Air) (mW cm^{-2})**	**Operating conditions**	**Ref.**
**FeCo-NCH**	**PAP-TP-100**	**PAP-TP-85**	**0.89**	**569.0**	**299.3**	**80 °C, 100% RH, 200 kPa**	**This work**
**Pt/C**	**PAP-TP-100**	**PAP-TP-85**	**0.86**	**750.7**	**508.1**	**80 °C, 100% RH, 100 kPa**	**This work**
Fe/N/C nanotubes	aQAPS-S_{14}	aQAPS-S_8	0.93	485		60 °C, 100% RH	21
Fe-NMG	AS-4	Tokuyama A201	0.83	218		60 °C, 100% RH	22
Fe-Co-N-C	ETFE-RG	RG-LDPE	0.76	420		60 °C, 52% RH	23
SNBC12	FAA-3-Br	FAA-3-50	0.85	215		60 °C, 80% RH	24
Fe-N-C	ETFE-RG	HDPE-AEM	0.84	2050	~1000	80 °C, 200 kPa	25
$MnCo_2O_4$/C	QAPPT	QAPPT	0.84	1200		80 °C, 100% RH, 100 kPa	26
FeCN-S-800	Fumion FAA-3	Fumapem FAA-3	0.76	125		50 °C	27
$Fe_{0.5}$-N-C	I2, Acta S.P.A.	A901-Tokuyama	0.85	504		50 °C, 100% RH, 150 kPa	28
MCS	aQAPS-S_{14}	aQAPS-S_8	0.85	1100		60 °C, 50% RH, 100 kPa	29
NFC@Fe/Fe_3C-9	FAA-3-SOLUT-10	Fumapem FAA-3	0.87	273		60 °C, 100% RH, 250 kPa	30
Fe/Ce-NCNW	FLN-55	m-TPN	0.915	500		80 °C, 100% RH	31
Co@G/C	I2	A201	0.8	412		60 °C, 100% RH	32
Co-N-CDC/CNT_mel	HMT-PMBI	HMT-PMBI	0.82	577		60 °C, 100% RH, 200 kPa	33
Co-NC	FAA-3	FAA-3	0.904	271		60 °C, 100% RH	34
Fe-N-Gra	HMT-PMBI	HMT-PMBI	0.77	243		60 °C, 100% RH	35

Fe/IL-PAN-A1000	HMT-PMBI	HMT-PMBI	0.74	289		60 °C, 100% RH, 200 kPa	36
Zn/Fe_{SA}-PC/950/HN_3	FAA-3	FAA-3-20	0.87	352		60 °C, 100% RH	37
FeCoNC-at	HMT-PMBI	HMT-PMBI	0.829	415		60 °C, 100% RH, 200 kPa	38
Fe-N-C	HMT-PMBI	HMT-PMBI	0.89	220		60 °C, 80% RH	39
FeN-SiCDC	HMT-PMBI	HMT-PMBI	-	356		60 °C, 82% RH, 200 kPa	40
FeCoN-MWCNT	FAA-3	VMFAA-3-10-rf	0.86	692	~420	60 °C, 100 kPa	41
CoFe-N-CDC/CNT	ETEE	ETFE-BTMA	0.83	1120	800	60 °C, 100% RH	42
PBC/900/M	FAA-3	FAA-3-20	0.862	658		60 °C, 100% RH	43
Fe/Co/IL-CNF-800b	-	HMT-PMBI	0.859	195		60 °C, 80% RH, 200 kPa	44
FeN_x-CNTs	-	PVA-0.8PQVBC40%	0.94	1150		60 °C, 200 kPa	45
Co_3N/C	QAPPT	QAPPT	0.862	700		80 °C, 100% RH	46
Fe-N-MPC	HMT-PMBI	HMT-PMBI	0.89	473		60 °C, 50% RH, 200 kPa	47
CoFe-N-OMC/CNT	AP2-INN8–00-X	AF2-HLE8-10-X	0.84	336		65 °C, 71% RH, 200 kPa	48
MPF/Fe	AP2-INN8–00-X	AF2-HLE8-10-X	0.81	347		65 °C, 65% RH, 200 kPa	49

Supplementary Table 5. Comparison of the site density (SD) and TOF performances for our electrocatalysts and reported PGM-free cathode electrocatalysts.

Catalysts	SD (site g^{-1})	TOF (e$^-$ s^{-1} site^{-1})	Ref.
**FeCo-NCH**	**7.6×10^{19}**	**0.6 @ 0.90 V**	**This work**
**FeCo-NC**	**2.1×10^{19}**	**0.2 @ 0.90 V**	**This work**
Co_2/Fe–N@CHC	~	0.6 @ 0.90 V	14
C@PVI-(DFTPP)Fe-800	~	0.47 @ 0.90 V	50
*$Fe_{0.5}$NC-800	3.99×10^{19}	0.46 @ 0.90 V	51
SA-Fe-NC	~	0.15 @ 0.90 V	52
*PANI-Fe	~1×10^{20}	0.23 @ 0.90 V	53
TPI@Z8(SiO_2)-650-C	~	1.63 @ 0.80 V	54
Fe-N/C	7.2×10^{18}	1.6 @ 0.80 V	55
Fe–NC$^{\Delta\text{-DCDA}}$	4.69×10^{19}	0.13 @ 0.85 V	56
PAJ	~2×10^{19}	~0.7 @ 0.80 V	57
CNRS	~6×10^{19}	~0.2 @ 0.80 V	57
FeNC-CVD-750	1.92×10^{20}	0.8 @ 0.80 V	58

The * marks represent SD and TOF were calculated based on CO sorption and desorption while the others are based on the ASD measured by the nitrite stripping experiments.

Supplementary Table 6. Comparison of the Zn-air battery performances for our catalysts and various state-of-the-art cathode catalysts.

Catalysts	Open circuit potential /V	Power density /mW cm^{-2}	Specific capacity / mAh g_{Zn}^{-1} (@mA cm^{-2})	Ref.
**FeCo-NCH**	**1.45**	**414.5**	**809.3@20**	**This Work**
Co/CNWs/CNFs	1.46	304	823@10	59
Co/CNT	-	318	-	60
Fe_2Ni@NC	1.493	126	-	61
LDH-POF	1.45	185	-	62
$NiFeO_x$@VACNTs	-	194	-	63
(Co, Fe)$_3$N_R	-	234	-	64
Fe_2/Co_1-GNCL	-	218	-	65
$Zn_{0.4}Ni_{0.6}Co_2O_4$	1.48	109.1	-	66
1100-CNS	1.49	151	-	67
SA-Fe-NHPC	1.52	266.4	795.3@10	68
Co@hNCTs-800	1.45	149	746@10	69
3DOM Fe–N–C-900	1.45	235	768.3@20	70
NPS-G	1.37	151	835@10	71
Silk NC/KB	1.46	~91.2	~736@50	72
Cu ISAS/NC	-	280	898@20	73
T-CoNCNTs	1.40	~245	-	74
SCoNC	-	≈194	≈690@5	75
A-Co@CMK-3-D	1.51	162	780@5	76
N/E-HPC-900	1.49	192.7	801@10	77
FeP_x/Fe–N–C/NPC	-	-	739@20	78
$Mn_{0.9}Fe_{2.1}$C/NC	~1.5	160	635@5	79
Mn/Fe-HIB-MOF	~1.48	195	769@5	80
Fe/Co-N/S-Cs	1.395	102.63	-	81
PANI-GO-1000	-	69.5	520@10	82

FeCo@MNC	1.41	143	-	83
$NiCo_2O_4$-rGO	-	125.3	719.8@5	84
NP Co_3O_4/Fe@C_2N	-	186.3	790.1@10	85
CoNi-SAs/NC	1.45	101.4	750.9@20	86
$NiCo_2S_4$@g-C_3N_4-CNT	1.35	142	485.7@10	87
Co-POC	-	78	-	88.
Co-ACs@NC	1.38	105.3	897.1@20	89
NOGB-800	1.5	111.9	-	90
$NiIn_2S_4$/CNFs	1.46	110	729@5	91
CMO/NCNF	1.46	224	-	92
7.1%Cu-Co_2P@2D-NPC	1.4	52.5	736.8@10	93
FeCo-DHO/NCNTs	1.48	193	793@20	94
C-MOF-C_2-900	1.46	105	768@5	95
Cu_3P@NPPC	1.46	110.8	-	96
Fe-N_4 SAs/NPC	-	232	-	97
TD-CFs	1.46	-	555@10	98
$LaMnO_{3+\delta}$	1.43	200	-	99

Supplementary References

1. Wang J. *et al.* Design of N-coordinated dual-metal sites: A stable and active Pt-free catalyst for acidic oxygen reduction reaction. *J. Am. Chem. Soc.* **139**, 17281-17284 (2017).
2. Fang X., Jiao L., Yu S.-H. & Jiang H.-L. Metal–organic framework-derived FeCo-N-doped hollow porous carbon nanocubes for electrocatalysis in acidic and alkaline media. *ChemSusChem* **10**, 3019-3024 (2017).
3. Gong S., Wang C., Jiang P., Hu L., Lei H. & Chen Q. Designing highly efficient dual-metal single-atom electrocatalysts for the oxygen reduction reaction inspired by biological enzyme systems. *J. Mater. Chem. A* **6**, 13254-13262 (2018).
4. Zhang D. *et al.* Isolated Fe and Co dual active sites on nitrogen-doped carbon for a highly efficient oxygen reduction reaction. *Chem. Commun.* **54**, 4274-4277 (2018).
5. Zhao R. *et al.* Puffing up energetic metal–organic frameworks to large carbon networks with hierarchical porosity and atomically dispersed metal sites. *Angew. Chem., Int. Ed.* **58**, 1975-1979 (2019).
6. Ren W. *et al.* Isolated diatomic Ni-Fe metal–nitrogen sites for synergistic electroreduction of CO_2. *Angew. Chem., Int. Ed.* **58**, 6972-6976 (2019).
7. Lu Z. *et al.* An isolated zinc–cobalt atomic pair for highly active and durable oxygen reduction. *Angew. Chem., Int. Ed.* **58**, 2622-2626 (2019).
8. Xiao M. *et al.* Climbing the apex of the ORR volcano plot via binuclear site construction: Electronic and geometric engineering. *J. Am. Chem. Soc.* **141**, 17763-17770 (2019).
9. Chen L. *et al.* Synergistic effect between atomically dispersed Fe and Co metal sites for enhanced oxygen reduction reaction. *J. Mater. Chem. A* **8**, 4369-4375 (2020).
10. Zhou Y. *et al.* Revealing of active sites and catalytic mechanism in N-coordinated Fe, Ni dual-doped carbon with superior acidic oxygen reduction than single-atom catalyst. *J. Phys. Chem. Lett.* **11**, 1404-1410 (2020).
11. Hu B. *et al.* Atomic Co/Ni dual sites with N/P-coordination as bifunctional oxygen electrocatalyst for rechargeable zinc-air batteries. *Nano Res.* **14**, 3482-3488 (2021).
12. Zhu Z. *et al.* Coexisting single-atomic Fe and Ni sites on hierarchically ordered porous carbon as a highly efficient ORR electrocatalyst. *Adv. Mater.* **32**, 2004670 (2020).
13. Zhu W. *et al.* Enhanced CO_2 electroreduction on neighboring Zn/Co monomers by electronic effect. *Angew. Chem., Int. Ed.* **59**, 12664-12668 (2020).
14. Wang Z. *et al.* Atomically dispersed Co_2–N_6 and Fe–N_4 costructures boost oxygen reduction reaction in both alkaline and acidic media. *Adv. Mater.* **33**, 2104718 (2021).
15. Cheng H. *et al.* Atomically dispersed Ni/Cu dual sites for boosting the CO_2 reduction reaction. *ACS Catal.* **11**, 12673-12681 (2021).
16. Li Y. *et al.* Synergistic effect of atomically dispersed Ni–Zn pair sites for enhanced CO_2 electroreduction. *Adv. Mater.* **33**, 2102212 (2021).
17. Jiao L. *et al.* Non-bonding interaction of neighboring Fe and Ni single-atom pairs on MOF-derived N-doped carbon for enhanced CO_2 electroreduction. *J. Am. Chem. Soc.* **143**, 19417-19424 (2021).
18. Zeng Z. *et al.* Orbital coupling of hetero-diatomic nickel-iron site for bifunctional electrocatalysis of CO_2 reduction and oxygen evolution. *Nat. Commun.* **12**, 4088 (2021).
19. Liang Z., Song L., Sun M., Huang B. & Du Y. Tunable CO/H_2 ratios of electrochemical reduction of CO_2 through the Zn-Ln dual atomic catalysts. *Sci. Adv.* **7**, eabl4915 (2021).

20. Feng M. *et al.* Well-defined Fe–Cu diatomic sites for efficient catalysis of CO_2 electroreduction. *J. Mater. Chem. A* **9**, 23817-23827 (2021).

21. Ren H. *et al.* Fe/N/C nanotubes with atomic Fe sites: A highly active cathode catalyst for alkaline polymer electrolyte fuel cells. *ACS Catal.* **7**, 6485-6492 (2017).

22. Hossen M. M., Artyushkova K., Atanassov P. & Serov A. Synthesis and characterization of high performing Fe-N-C catalyst for oxygen reduction reaction (ORR) in alkaline exchange membrane fuel cells. *J. Power Sources* **375**, 214-221 (2018).

23. Osmieri L., Zafferoni C., Wang L., Monteverde Videla A. H. A., Lavacchi A. & Specchia S. Polypyrrole-derived Fe−Co−N−C catalyst for the oxygen reduction reaction: Performance in alkaline hydrogen and ethanol fuel cells. *ChemElectroChem* **5**, 1954-1965 (2018).

24. Kim M.-J. *et al.* Biomass-derived air cathode materials: Pore-controlled S, N-Co-doped carbon for fuel cells and metal–air batteries. *ACS Catal.* **9**, 3389-3398 (2019).

25. Peng X., Omasta T. J., Magliocca E., Wang L., Varcoe J. R. & Mustain W. E. Nitrogen-doped carbon–CoO_x nanohybrids: A precious metal free cathode that exceeds 1.0 W cm^{-2} peak power and 100 h life in anion-exchange membrane fuel cells. *Angew. Chem., Int. Ed.* **58**, 1046-1051 (2019).

26. Yang Y. *et al.* High-loading composition-tolerant Co–Mn spinel oxides with performance beyond 1 W/cm^2 in alkaline polymer electrolyte fuel cells. *ACS Energy Lett.* **4**, 1251-1257 (2019).

27. Huang H.-C. *et al.* Nanostructured cementite/ferrous sulfide encapsulated carbon with heteroatoms for oxygen reduction in alkaline environment. *ACS Sustainable Chem. Eng.* **7**, 3185-3194 (2019).

28. Lee S. H. *et al.* Design principle of Fe–N–C electrocatalysts: How to optimize multimodal porous structures? *J. Am. Chem. Soc.* **141**, 2035-2045 (2019).

29. Wang Y. *et al.* Synergistic Mn-Co catalyst outperforms Pt on high-rate oxygen reduction for alkaline polymer electrolyte fuel cells. *Nat. Commun.* **10**, 1506 (2019).

30. Karuppannan M., Park J. E., Bae H. E., Cho Y.-H. & Kwon O. J. A nitrogen and fluorine enriched Fe/Fe_3C@C oxygen reduction reaction electrocatalyst for anion/proton exchange membrane fuel cells. *Nanoscale* **12**, 2542-2554 (2020).

31. Li J.-C. *et al.* Stabilizing single-atom iron electrocatalysts for oxygen reduction via ceria confining and trapping. *ACS Catal.* **10**, 2452-2458 (2020).

32. Sharma M. *et al.* Work function-tailored graphene via transition metal encapsulation as a highly active and durable catalyst for the oxygen reduction reaction. *Energy Environ. Sci.* **12**, 2200-2211 (2019).

33. Lilloja J. *et al.* Cathode catalysts based on cobalt- and nitrogen-doped nanocarbon composites for anion exchange membrane fuel cells. *ACS Appl. Energy Mater.* **3**, 5375-5384 (2020).

34. Im K., Kim D., Jang J.-H., Kim J. & Yoo S. J. Hollow-sphere Co-NC synthesis by incorporation of ultrasonic spray pyrolysis and pseudomorphic replication and its enhanced activity toward oxygen reduction reaction. *Appl. Catal., B* **260**, 118192 (2020).

35. Sibul R. *et al.* Iron- and nitrogen-doped graphene-based catalysts for fuel cell applications. *ChemElectroChem* **7**, 1739-1747 (2020).

36. Mooste M. *et al.* Electrospun polyacrylonitrile-derived Co or Fe containing nanofibre catalysts for oxygen reduction reaction at the alkaline membrane fuel cell cathode. *ChemCatChem* **12**, 4568-4581 (2020).

37. Kim H. S. *et al.* Waste pig blood-derived 2D Fe single-atom porous carbon as an efficient electrocatalyst for zinc–air batteries and AEMFCs. *Appl. Surf. Sci.* **563**, 150208 (2021).
38. Kisand K. *et al.* Transition metal-containing nitrogen-doped nanocarbon catalysts derived from 5-methylresorcinol for anion exchange membrane fuel cell application. *J. Colloid Interface Sci.* **584**, 263-274 (2021).
39. Lilloja J. *et al.* Mesoporous iron-nitrogen co-doped carbon material as cathode catalyst for the anion exchange membrane fuel cell. *J. Power Sources Adv.* **8**, 100052 (2021).
40. Ratso S. *et al.* Non-precious metal cathodes for anion exchange membrane fuel cells from ball-milled iron and nitrogen doped carbide-derived carbons. *Renewable Energy* **167**, 800-810 (2021).
41. Kumar Y. *et al.* Bifunctional oxygen electrocatalysis on mixed metal phthalocyanine-modified carbon nanotubes prepared via pyrolysis. *ACS Appl. Mater. Interfaces* **13**, 41507-41516 (2021).
42. Lilloja J. *et al.* Transition-metal- and nitrogen-doped carbide-derived carbon/carbon nanotube composites as cathode catalysts for anion-exchange membrane fuel cells. *ACS Catal.* **11**, 1920-1931 (2021).
43. Lee J. *et al.* Atomic-scale engineered Fe single-atom electrocatalyst based on waste pig blood for high-performance AEMFCs. *ACS Sustainable Chem. Eng.* **9**, 7863-7872 (2021).
44. Sokka A. *et al.* Iron and cobalt containing electrospun carbon nanofibre-based cathode catalysts for anion exchange membrane fuel cell. *Int. J. Hydrogen Energy* **46**, 31275-31287 (2021).
45. He Q. *et al.* Polymer-coating-induced synthesis of FeN_x enriched carbon nanotubes as cathode that exceeds 1.0 W cm^{-2} peak power in both proton and anion exchange membrane fuel cells. *J. Power Sources* **489**, 229499 (2021).
46. Zeng R. *et al.* Nonprecious transition metal nitrides as efficient oxygen reduction electrocatalysts for alkaline fuel cells. *Sci. Adv.* **8**, eabj1584 (2022).
47. Lilloja J. *et al.* Transition metal and nitrogen-doped mesoporous carbons as cathode catalysts for anion-exchange membrane fuel cells. *Appl. Catal., B* **306**, 121113 (2022).
48. Lilloja J. *et al.* Cobalt-, iron- and nitrogen-containing ordered mesoporous carbon-based catalysts for anion-exchange membrane fuel cell cathode. *Electrochim. Acta* **439**, 141676 (2023).
49. Akula S. *et al.* Transition metal (Fe, Co, Mn, Cu) containing nitrogen-doped porous carbon as efficient oxygen reduction electrocatalysts for anion exchange membrane fuel cells. *Chem. Eng. J.* **458**, 141468 (2023).
50. Zhao Y.-M. *et al.* Design and preparation of Fe–N_5 catalytic sites in single-atom catalysts for enhancing the oxygen reduction reaction in fuel cells. *ACS Appl. Mater. Interfaces* **12**, 17334-17342 (2020).
51. Luo F. *et al.* Accurate evaluation of active-site density (SD) and turnover frequency (TOF) of PGM-free metal–nitrogen-doped carbon (MNC) electrocatalysts using CO cryo adsorption. *ACS Catal.* **9**, 4841-4852 (2019).
52. Liang X. *et al.* Two types of single-atom FeN_4 and FeN_5 electrocatalytic active centers on N-doped carbon driving high performance of the SA-Fe-NC oxygen reduction reaction catalyst. *Chem. Mater.* **33**, 5542-5554 (2021).
53. Luo F. *et al.* Kinetic diagnostics and synthetic design of platinum group metal-free electrocatalysts for the oxygen reduction reaction using reactivity maps and site utilization descriptors. *J. Am. Chem. Soc.* **144**, 13487-13498 (2022).

54. Wan X. *et al.* Fe–N–C electrocatalyst with dense active sites and efficient mass transport for high-performance proton exchange membrane fuel cells. *Nat. Catal.* **2**, 259-268 (2019).
55. Malko D., Kucernak A. & Lopes T. *In situ* electrochemical quantification of active sites in Fe–N/C non-precious metal catalysts. *Nat. Commun.* **7**, 13285 (2016).
56. Mehmood A. *et al.* High loading of single atomic iron sites in Fe–NC oxygen reduction catalysts for proton exchange membrane fuel cells. *Nat. Catal.* **5**, 311-323 (2022).
57. Primbs M. *et al.* Establishing reactivity descriptors for platinum group metal (PGM)-free Fe–N–C catalysts for PEM fuel cells. *Energy Environ. Sci.* **13**, 2480-2500 (2020).
58. Jiao L. *et al.* Chemical vapour deposition of Fe–N–C oxygen reduction catalysts with full utilization of dense Fe–N_4 sites. *Nat. Mater.* **20**, 1385-1391 (2021).
59. Xia C. *et al.* Electrospinning synthesis of self-standing cobalt/nanocarbon hybrid membrane for long-life rechargeable zinc–air batteries. *Adv. Funct. Mater.* **31**, 2105021 (2021).
60. Zhou T. *et al.* Nanopore confinement of electrocatalysts optimizing triple transport for an ultrahigh-power-density zinc–air fuel cell with robust stability. *Adv. Mater.* **32**, 2003251 (2020).
61. Zhu J. *et al.* A triphasic bifunctional oxygen electrocatalyst with tunable and synergetic interfacial structure for rechargeable Zn-air batteries. *Adv. Energy Mater.* **10**, 1903003 (2020).
62. Zhao C.-X. *et al.* Multiscale construction of bifunctional electrocatalysts for long-lifespan rechargeable zinc–air batteries. *Adv. Funct. Mater.* **30**, 2003619 (2020).
63. Yan Y. *et al.* Bifunctional nickel ferrite-decorated carbon nanotube arrays as free-standing air electrode for rechargeable Zn–air batteries. *J. Mater. Chem. A* **8**, 5070-5077 (2020).
64. Deng, YP., Jiang, Y., Liang, R. *et al.* Dynamic electrocatalyst with current-driven oxyhydroxide shell for rechargeable zinc-air battery. *Nat Commun* **11**, 1952 (2020).
65. Wei Y.-S. *et al.* Fabricating dual-atom iron catalysts for efficient oxygen evolution reaction: A heteroatom modulator approach. *Angew. Chem., Int. Ed.* **59**, 16013-16022 (2020).
66. Wang X.-T., Ouyang T., Wang L., Zhong J.-H. & Liu Z.-Q. Surface reorganization on electrochemically-induced Zn–Ni–Co spinel oxides for enhanced oxygen electrocatalysis. *Angew. Chem., Int. Ed.* **59**, 6492-6499 (2020).
67. Pei Z. *et al.* Texturing *in situ*: N,S-enriched hierarchically porous carbon as a highly active reversible oxygen electrocatalyst. *Energy Environ. Sci.* **10**, 742-749 (2017).
68. Chen G. *et al.* Zinc-mediated template synthesis of Fe-N-C electrocatalysts with densely accessible Fe-N_x active sites for efficient oxygen reduction. *Adv. Mater.* **32**, 1907399 (2020).
69. Zhou Q. *et al.* Template-guided synthesis of Co nanoparticles embedded in hollow nitrogen doped carbon tubes as a highly efficient catalyst for rechargeable Zn-air batteries. *Nano Energy* **71**, 104592 (2020).
70. Zhang X. *et al.* Atomically dispersed hierarchically ordered porous Fe–N–C electrocatalyst for high performance electrocatalytic oxygen reduction in Zn-air battery. *Nano Energy* **71**, 104547 (2020).
71. Zheng X. *et al.* N-, P-, and S-doped graphene-like carbon catalysts derived from onium salts with enhanced oxygen chemisorption for Zn-air battery cathodes. *Appl. Catal., B* **241**, 442-451 (2019).

72. Wang C. *et al.* Silk-derived highly active oxygen electrocatalysts for flexible and rechargeable Zn–air batteries. *Chem. Mater.* **31**, 1023-1029 (2019).
73. Yang Z. *et al.* Directly transforming copper (I) oxide bulk into isolated single-atom copper sites catalyst through gas-transport approach. *Nat. Commun.* **10**, 3734 (2019).
74. Xu W. *et al.* An advanced zinc air battery with nanostructured superwetting electrodes. *Energy Stor. Mater* **17**, 358-365 (2019).
75. Wu J. *et al.* Densely populated isolated single Co-N site for efficient oxygen electrocatalysis. *Adv. Energy Mater.* **9**, 1900149 (2019).
76. Lyu X. *et al.* Atomic cobalt on defective bimodal mesoporous carbon toward efficient oxygen reduction for zinc–air batteries. *Small Methods* **3**, 1800450 (2019).
77. Peng X. *et al.* Hierarchically porous carbon plates derived from wood as bifunctional ORR/OER electrodes. *Adv. Mater.* **31**, 1900341 (2019).
78. Qin Q., Jang H., Li P., Yuan B., Liu X. & Cho J. A tannic acid–derived N-, P-codoped carbon-supported iron-based nanocomposite as an advanced trifunctional electrocatalyst for the overall water splitting cells and zinc–air batteries. *Adv. Energy Mater.* **9**, 1803312 (2019).
79. Lin C. *et al.* Long-life rechargeable Zn air battery based on binary metal carbide armored by nitrogen-doped carbon. *ACS Appl. Energy Mater.* **2**, 1747-1755 (2019).
80. Shinde S. S. *et al.* Unveiling dual-linkage 3D hexaiminobenzene metal–organic frameworks towards long-lasting advanced reversible Zn–air batteries. *Energy Environ. Sci.* **12**, 727-738 (2019).
81. Li C., Liu H. & Yu Z. Novel and multifunctional inorganic mixing salt-templated 2D ultrathin Fe/Co-N/S-carbon nanosheets as effectively bifunctional electrocatalysts for Zn-air batteries. *Appl. Catal., B* **241**, 95-103 (2019).
82. Huang Y. *et al.* Atomic modulation and structure design of carbons for bifunctional electrocatalysis in metal–air batteries. *Adv. Mater.* **31**, 1803800 (2019).
83. Li C., Wu M. & Liu R. High-performance bifunctional oxygen electrocatalysts for zinc-air batteries over mesoporous Fe/Co-N-C nanofibers with embedding FeCo alloy nanoparticles. *Appl. Catal., B* **244**, 150-158 (2019).
84. Li Y. *et al.* Shape-controlled synthesis of $NiCo_2O_4$-rGO as bifunctional electrocatalyst for Zn-air battery. *ChemElectroChem* **6**, 4429-4436 (2019).
85. Kim J. *et al.* Synergistic coupling derived cobalt oxide with nitrogenated holey two-dimensional matrix as an efficient bifunctional catalyst for metal–air batteries. *ACS Nano* **13**, 5502-5512 (2019).
86. Han X. *et al.* Atomically dispersed binary Co-Ni sites in nitrogen-doped hollow carbon nanocubes for reversible oxygen reduction and evolution. *Adv. Mater.* **31**, 1905622 (2019).
87. Han X. *et al.* Identifying the activation of bimetallic sites in $NiCo_2S_4$@g-C_3N_4-CNT hybrid electrocatalysts for synergistic oxygen reduction and evolution. *Adv. Mater.* **31**, 1808281 (2019).
88. Li B.-Q. *et al.* Framework-porphyrin-derived single-atom bifunctional oxygen electrocatalysts and their applications in Zn–air batteries. *Adv. Mater.* **31**, 1900592 (2019).
89. Han X. *et al.* Generation of nanoparticle, atomic-cluster, and single-atom cobalt catalysts from zeolitic imidazole frameworks by spatial isolation and their use in zinc–air batteries. *Angew. Chem., Int. Ed.* **58**, 5359-5364 (2019).

90. Hu Q. *et al.* Trifunctional electrocatalysis on dual-doped graphene nanorings–integrated boxes for efficient water splitting and Zn–air batteries. *Adv. Energy Mater.* **9**, 1803867 (2019).
91. Fu G., Wang Y., Tang Y., Zhou K., Goodenough J. B. & Lee J.-M. Superior oxygen electrocatalysis on nickel indium thiospinels for rechargeable Zn–air batteries. *ACS Mater. Lett* **1**, 123-131 (2019).
92. Chen X. *et al.* Spinel oxide nanoparticles embedded in nitrogen-doped carbon nanofibers as a robust and self-standing bifunctional oxygen cathode for Zn–air batteries. *J. Mater. Chem. A* **7**, 24868-24876 (2019).
93. Diao L. *et al.* Electronic reconfiguration of Co_2P induced by Cu doping enhancing oxygen reduction reaction activity in zinc–air batteries. *J. Mater. Chem. A* **7**, 21232-21243 (2019).
94. Wu M. *et al.* Fe/Co double hydroxide/oxide nanoparticles on N-doped CNTs as highly efficient electrocatalyst for rechargeable liquid and quasi-solid-state zinc–air batteries. *Adv. Energy Mater.* **8**, 1801836 (2018).
95. Zhang M., Dai Q., Zheng H., Chen M. & Dai L. Novel MOF-derived Co@N-C bifunctional catalysts for highly efficient Zn–air batteries and water splitting. *Adv. Mater.* **30**, 1705431 (2018).
96. Wang R., Dong X.-Y., Du J., Zhao J.-Y. & Zang S.-Q. MOF-derived bifunctional Cu_3P nanoparticles coated by a N,P-codoped carbon shell for hydrogen evolution and oxygen reduction. *Adv. Mater.* **30**, 1703711 (2018).
97. Pan Y. *et al.* A bimetallic Zn/Fe polyphthalocyanine-derived single-atom Fe-N_4 catalytic site:A superior trifunctional catalyst for overall water splitting and Zn–air batteries. *Angew. Chem., Int. Ed.* **57**, 8614-8618 (2018).
98. Wang L. *et al.* Nitrogen, fluorine, and boron ternary doped carbon fibers as cathode electrocatalysts for zinc–air batteries. *Small* **14**, 1800737 (2018).
99. Kuai L. *et al.* Mesoporous $LaMnO_{3+\delta}$ perovskite from spray−pyrolysis with superior performance for oxygen reduction reaction and Zn−air battery. *Nano Energy* **43**, 81-90 (2018).
